# Synthesis Characterization and Antibacterial, Antifungal Activity of N-(Benzyl Carbamoyl or Carbamothioyl)-2-hydroxy Substituted Benzamide and 2-Benzyl Amino-Substituted Benzoxazines

**DOI:** 10.1155/2013/436397

**Published:** 2013-10-31

**Authors:** Tyson Belz, Saleh Ihmaid, Jasim Al-Rawi, Steve Petrovski

**Affiliations:** School of Pharmacy and Applied Science, La Trobe University, P.O. Box 199, Bendigo, UIC 3552, Australia

## Abstract

New N-(benzyl carbamothioyl)-2-hydroxy substituted benzamides **13**, **20**, and **21** were synthesized using sodium bicarbonate and benzyl amine with 2-thioxo-substituted-1,3-benzoxazines **6, 10a, b, 11c**, and **12a–n**. The 2-thioxo-substituted-1,3-oxazines **6, 10a-b, 11d 12a–n**, and **26** were converted to the corresponding 2-methylthio-substituted-1,3-oxazines **14a–l** and **24** which were then converted to 2-benzyl amino-substituted-benzoxazines **15a–i** by refluxing with benzylamine. Products **15a, b, e, f**, and **g** were also synthesized by boiling the corresponding N-(benzyl carbamothioyl)-2-hydroxy substituted benzamides **13a, b, f, l**, and **m** in acetic acid. 2-Oxo-substituted-1,3-benzoxazines **22** and **25** were prepared by treating the corresponding 2-methylthio-substituted-1,3-oxazines **14** and **24** with dilute HCl. The N-(benzyl carbamoyl)-2-hydroxy substituted benzamide **23** was synthesized from the reaction of 2-oxo-substituted-1,3-benzoxazine **22** with benzylamine. The new products were characterized using IR, ^1^H, and ^13^C NMR in addition to microanalysis. Selected compounds were tested in vitro for antibacterial and antifungi activity and the most active compounds were found to be the 4-(substituted-benzylamino)-2-hydroxy benzoic acids **9a** and **d** (*M. chlorophenolicum*, MIC 50 and 25 *µ*gm L^−1^, resp.), *N*1, *N*3-bis (benzyl carbamothioyl)-4,6-dihydroxy-substituted phthalamides **20a** and **20c** (*B. subtilis* MIC 12.5, 50 *µ*gm L^−1^, resp.) and **21** (*M. chlorophenolicum*, MIC 50 *µ*gm L^−1^).

## 1. Introduction

The search for new antibacterial compounds is a challenging task as bacteria are continuously developing resistance to antimicrobial compounds; however, infections due to such bacterial strains are infrequent although potentially fatal [1–3]. This ongoing problem has resulted in the search for newer, more effective antibacterial compounds [1–3].

**Scheme 1:**
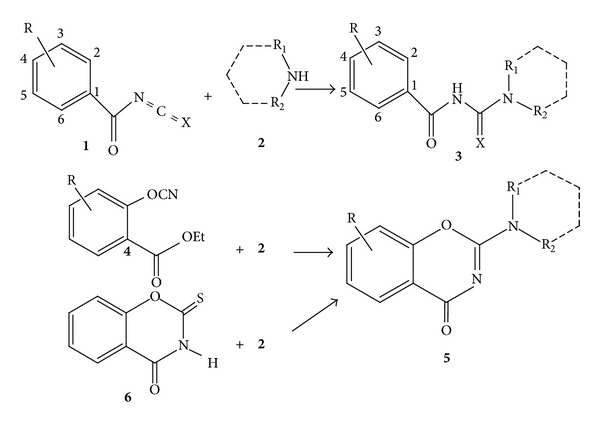
Previous synthesis of urea or thiourea **3** (X=O or S) and 2-amino benzo1,3-oxazine **5**.

Urea, thiourea **3** (X=O or S), and benzo-1,3-oxazine compounds **5** and **6** (Scheme 1) have been shown to possess antibacterial and antifungal properties [4–11]. The benzyl thiourea analogue **3** has been reported to show activity against Gram-positive bacteria [12].

The N-benzoyl-2-hydroxybenzamides [13] are important pharmacophores for antibacterial activity in which the 2-hydroxy group (hydrogen bonding donor) contributes to the activity, the imide linker (preferred) or urea linker retains activity and free NH is required for high activity.

The Topliss method [14] was used in the optimization of salicylic acid derivatives for potential use as antibacterial agents. The employment and analysis of physicochemical parameters and molecular electronic surfaces which highlight the electronic, lipophilic, and steric features may be useful guidelines in the continuous search for new, more effective 3-amino-salicylic acid analogs. The synthesis of the urea or thiourea product (**3**, Scheme 1) was previously achieved from the reaction of benzoylisocyanate or benzoylisothiocyanate 1 (X=O or S, resp.) with amines **2** [15–17].

The substitution R on the aromatic ring could be alkyl, alkoxy, –OC=OCH_3_ in positions 2, 3, 4, 5, and 6, and the R_1_-N-R_2_in product **3** could be aliphatic, aromatic, or cyclic amine substitutions. Limitation associated with this method is that R, the substitution on the aromatic ring, cannot be –OH or RN–, which is desirable for antimicrobial activity, particularly the hydroxy group [18].

Furthermore, the synthesis of 2-amino-substituted-1,3- benzoxazine 5 was achieved through the reaction of the corresponding amine **2** with ethyl 2-cyanobenzoate **4** [19–21]. However, again the limitation in this method is that the R in product **5** cannot be –OH or –NH_2_.

The reaction of substituted 2-thio-1,3-benzoxazine-4-one **6** (Scheme 1) with primary and secondary amines showed considerable interest in the literature.

**Scheme 2:**
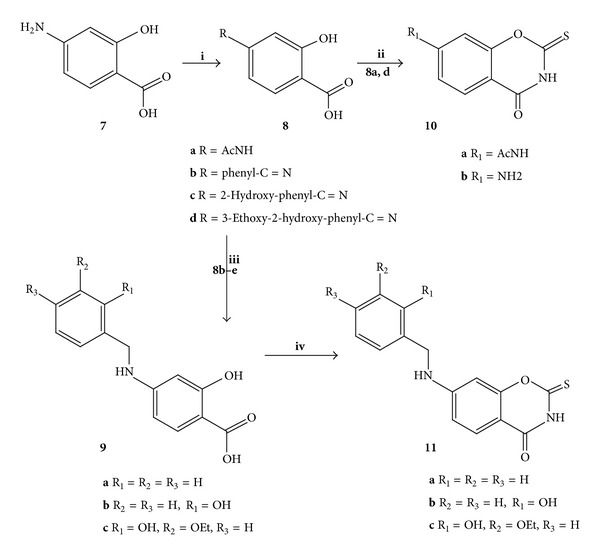
Synthesis of 7-N-substituted-1,3-oxazines **10-11** starting from 2-hydroxy-substituted benzoic acids **8** and **9**. Reaction conditions: (**i**) compound **a** (Ac)_2_O, **b** PhCH=O, **c** 2-hydroxy-C_6_H_4_-CH=O, and **d** 3-ethoxy-2-hydroxy-C_6_H_3_-CH=O, (**ii**) Ph_3_P(SCN)_2_ in CH_2_Cl_2_, (**iii**) NaBH_4_, and (**iv**) Ph_3_P(SCN)_2_ in CH_2_Cl_2_.

The secondary amine **2** (dimethyl, diethyl, and cyclic amines) reaction with 2-thioxo-2,3-substituted 1,3-benzoxazin-4-one **6** gave only 2-amino-substituted-1,3-benzoxazine **5** [19, 20, 22, 23]. Reactions with primary amines lead to the opening of the oxazine ring and produce thiourea type analogues (compound **3** R = 2-OH and R_1_NR_2_ = –HN-alkyl) while with NH_2_ CH_2_Ph gave **3** R = 2-OH and R_1_NR_2_ = NHCH_2_Ph mixed with 2-(benzyl amino)-4*H*-benz[*e*]-1,3-oxazin-4-one (5 R = H and R_1_NR_2_ = –NHCH_2_Ph [19, 20]. The above reaction produced low yields or a mixture of products **13a** and **15a** (Scheme 2) which was difficult to separate.

In this work, we developed simple general procedures for the synthesize of N-(benzyl carbamothioyl)-2-hydroxybenzamides **13**, **20**, and **21**, N-(benzyl carbamoyl)-2-hydroxy-substituted-benzamides **23**, and 2-(benzyl amino)-substituted-1,3-benzoxazin-4-one **15** and **26**. The antibacterial and antifungal activity was evaluated for a number of these products with the intension of producing novel products that can be used to eliminate problematic bacteria in the environmental and medical settings. These compounds could potentially result in novel antibiotic.

## 2. Results and Discussion

### 2.1. Chemistry

#### 2.1.1. Synthesis of Substituted 2-Hydroxy Aromatic Carboxylic Acids

5-Substituted-4,6-dihydroxybenzene-1,3-dicarboxylic acids **16a–c**, and 2,3-dihydroxybenzene-1,4-dicarboxylic acid **17** were prepared by carboxylation of 2-substituted-1,3-hydroxy-benzene and 1,2-dihydroxy-benzene, respectively, according to the previously reported method [24]. Compound **7** was selectively acetylated in the presence of NaOH and acetic anhydride with the reaction maintained at pH 6-7 in accordance with the reported procedure [25–27] to give the acid **8a** an excellent yield (82%) (Scheme 2). 

Allowing compound **7** to react with substituted benzaldehyde gave the corresponding Schiff base **8b-c** (Scheme 2) [28–33]. Reduction of Schiff base **8b-c** with sodium borohydride gave 4-(substituted-benzylamino)-2-hydroxybenzoic acids **9a–c** with high yield (73–85%). The structures of the prepared acids were confirmed using mp, IR, ^1^H, and ^13^CNMR which was in good agreement with the previously reported physical and spectroscopy data [26–28].

#### 2.1.2. Synthesis of Substituted 2-Thio-substituted-benzoxazines

**Scheme 3:**
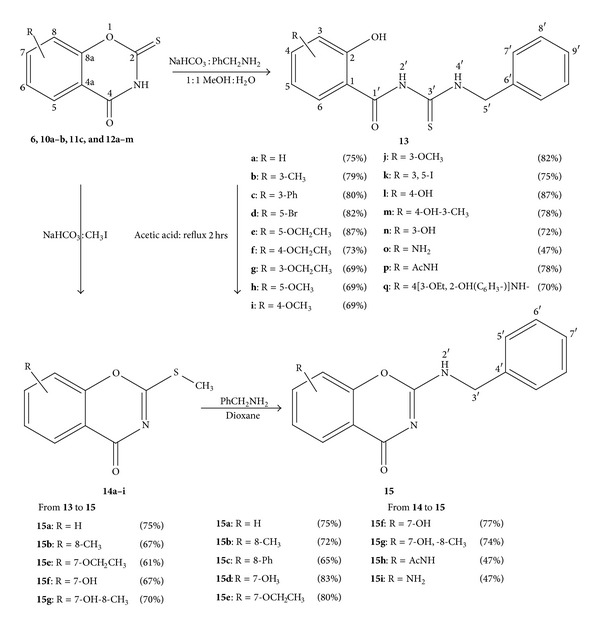
Synthesis of *N*-(benzyl carbamothioyl)-2-hydroxy-substituted benzamide **13**, substituted 2-benzylamino-1,3-benzoxazines **15**.

The 2-thio-substituted-1,3-benzoxazines **6**, **10**, **11**, **12** (**a** R = 8-CH_3_, **b** R = 8-Ph, **c** R = 6-Br, **d** R = 6-OCH_2_CH_3_, **e** R = 7-OCH_2_CH_3_, **f** R = 8-OCH_2_CH_3_, **g** R = 6-OCH_3_, **h** R = 7-OCH_3_, **i** R = 8-OCH_3_, **j** R = 6,8-I, **k** R = 7-OH, **l** R = 7-OH,-8-CH_3_, and **m** R = 8-OH), **18**, and **19** were synthesized from the reaction of the substituted 2-hydroxy benzoic acid with freshly prepared Ph_3_P(SCN)_2_ (Schemes 2 and 3) following the reported conditions [22, 34] with some modifications (see Experimental). It is worth noting that the reaction of 4-benzylideneamino-2-hydroxybenzoic acid **8d** formed the corresponding 7-(benzylidene)amino-2-thio-1,3-benzoxazines which were hydrolysed during the isolation of the product and gave 7-amino-2-thio-1,3-benzoxazines **10b**. However, the reaction of 4-amino-2-hydroxybenzoic acid **7** with the freshly prepared Ph_3_P(SCN)_2_ failed to produce the expected product **10b** however gave a complex, unidentifiable molecule containing a triphenylphosphene group attached to the oxazine product.

7-(Substituted benzylamino)-2-thioxo-2,3-dihydro-4*H*-1,3-benzoxazin-4-one **11a–c** were synthesized from the reaction of 4-(substituted-benzylamino)-2-hydroxybenzoic acids **9a–c** with the freshly prepared Ph_3_P(SCN)_2_ [22, 32] (Scheme 2). The structures of the new products **11a–c** were confirmed using ^1^H, and ^13^CNMR and microanalysis.

Similarly, the substituted-2-thioxo-1,3-benzoxazin-4-one **6** and **12b–n** prepared using a previously reported [22, 23, 33] method gave products with identical physical and spectroscopic data.

The benz-bis-(1,3-oxazine) **18a–c** and **19** were prepared from the reaction of dihydroxy-dicarboxybenzoic acids **16a–c** and **17** with the freshly prepared Ph_3_P(SCN)_2_ [22, 32] according to the previously reported method [34] for the synthesis of **18c** with some modification to improve the yield.

**Scheme 4:**
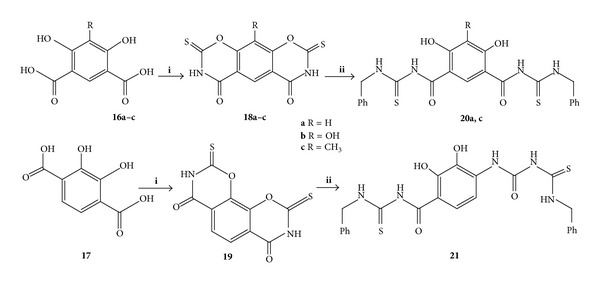
Synthesis of substituted-*N*,*N*-bis(benzyl carbamothioyl)-dihydroxy-iso and tetra phthalamides **20a**, **c**, and **21**. Reaction conditions: (**i**) Ph_3_P(SCN)_2_ in CH_2_Cl_2_ and (**ii**) NaHCO_3_, PhCH_2_NH_2_.

The structures of the new dibenzoxines products **18a.b** and **19** were confirmed using ^1^H and ^13^CNMR and microanalysis (Scheme 4).

#### 2.1.3. Synthesis of the 2-Methylthio-substituted-1,3-oxazines

2-Methylthio-substituted-1,3-oxazines **14a–i** (**a** R = H, **b** R = 8-CH_3_, **c** R = 8-Ph, **d** R = 7-OCH_3_, **e** R = 7-OCH_2_CH_3_, **f** R = 7-OH, **g** R = 7-OH,-8-CH_3_, **h** R = AcNH, and I R = NH_2_) and **24** were prepared by the reaction of CH_3_I in the presence of NaHCO_3_ with substituted-1,3-oxazines **6**, **10a**,**b**, **11c**, and **12** (**a** R = 8-CH_3_, **b** R = 8-Ph, **e** R = 7-OCH_2_CH_3_, **h** R = 7-OCH_3_, **k** R = 7-OH, and **l** R = 7-OH,-8-CH_3_) according to the previously reported procedure [35]. Physical properties, IR, ^1^H NMR, and ^13^C NMR, collected for products **14a–I** were found to be identical to the reported data [35]. The structures of new product, **14 h**, **i**, and **24** were confirmed with analysis of the IR, ^1^H, and ^13^C NMR data.

Compounds **14a–i** and **24** were used in the synthesis of compounds **15a–I** and **26** with no further purification.

#### 2.1.4. Synthesis of Benzyl Thiourea **13a–q**, **20a**, **c**, and **21**

Initially, the equimolar reaction of compound **6** with benzylamine in dioxane was repeated according to the earlier reported method [19, 20], in which the mixture that was refluxed for 4 hours gave **13a** and was a 46% yield (Scheme 3). 

However, when the reaction was carried out using solvent free conditions, excess benzylamine was added directly to powered 2-thio-1,3-benzoxazine 6 and the mixture was left at room temperature for 2 days, the benzyl thiourea product **13a** had decreased yield (19%), and none of the cyclic analogue **15a** could be isolated. When 2-thio-1,3-benzoxazine **6** was allowed to react with excess benzylamine (4-fold) and the mixture was then heated to reflux in dioxane for 2 hours, the open product **13a** was again isolated in 18% yield.

To overcome this low yield and possible mixture formation, the reaction procedure was modified in which the benzoxazines **6**, **10a-b**, **11d**, and **12b–m** were mixed with NaHCO_3_ and suspended in 1 : 1 mixture of methanol, water and the mixture was then heated to 40°C for a few minutes. Excess (1.5-fold) benzyl amine was added dropwise at room temperature and left stirring for 4 hours. Products **13a–q** was isolated according to the general procedure B (see Experimental) and the yield were moderate to high (69–87%) (Scheme 3).

With slight modification to the procedure B, by altering the ratio of benzylamine to starting material (3 : 1) and the reaction time to 16 hours, the bis-oxazines **18a,c** and **19** were found to react in a similar fashion to give substituted bis(benzyl carbamothioyl) analogues **20a,c** (yield 63, 49%) and **21** (yield 63%) (Scheme 4).

The previously prepared N-(benzyl carbamothioyl)-2-hydroxybenzamide **13a** was characterized by comparison of its physical data (mp, IR,^1^H and ^13^C NMR spectra) with values found in the literature [12, 19, 20]. The structures of new benzyl thiourea compounds **13b–q**, **20a,c**, and **21** were confirmed using IR, ^1^H NMR and ^13^C NMR spectroscopy and microanalysis. The ^1^H NMR and IR spectra also showed a high correlation with the previously prepared benzyl thiourea **13a** [19, 20]. In the ^1^H NMR spectra, the CH_2_, H-5′ of the benzyl amine in compounds **13a–q**, **20a**,**c**, and **21** appeared as a doublet at ~*δ* 4.9 ppm and the 4′-NH, appeared as a triplet at ~*δ* 11.0 ppm in all cases. Assignment of the carbon-13 chemical shifts was made using the previous reported chemical shifts of **13a** [19, 20]. The ^1^H and ^13^C NMR spectra of the parent 2-thioxo-2*H*-benz[*e*]-1,3-oxazin-4(3*H*)-one **12** were also used to aid with structural identification. The simulated ^1^H and ^13^C NMR spectra using ChemDraw V12 ultra were also used as references to aid the analysis of the observed ^1^H and ^13^C NMR spectra of the new products.

#### 2.1.5. Synthesis of 2-Benzyl amino-(substituted)-benz-1,3-oxazines **15**

As mentioned earlier the reaction of the 2-thio-1,3-benzoxazine **6** with a primary amine did not consistently give the cyclic product **13a** [19, 20].

**Scheme 5:**
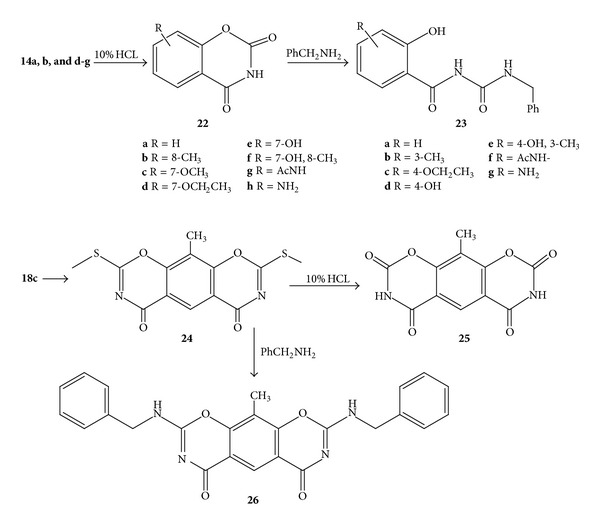
Synthesis of 2-dione-1,3-benzoxazines **22a–I** and **25a,b** from the 2-methylthio-1,3-benzoxazines **14a,b**, **d–g**, and **24a-b**.

The reaction of 2-methylthio-(substituted)-1,3-benzoxazines **14** (**a** R = H, **b** R = 8-CH_3_, **c** R = 8-Ph, **d** R = 7-OCH_3_, **e** R = 7-OCH_2_CH_3_, **f** R = 7-OH, **g** R = 7-OH,-8-CH_3_, and **h** R = AcNH-) and **24** with excess (5-fold) benzyl amine according to the general procedure C gave 2-benzyl amino-substituted-1,3-benzoxazines **15a–h** and **26** with moderate to high yields 62–83% (Schemes 3 and 5). 

The reaction of benzylamine with 2-methylthio-benzoxazine took place with no trace of the thiourea analogue **13**.

Following their successful synthesis, many of the *N*-(benzyl carbamothioyl)-2-hydroxybenzamides **13a**, **b**, **f**, and **g** were then cyclised by refluxing in acetic acid for 2 hours according to the general procedure D (Scheme 3) and gave the corresponding 2-benzylamino-1,3-benzoxazines **15a**, **b**, **f**, and **g** fair to good yields.

Previously prepared 2-benzyl amino-1,3-benzoxazine **15a** was characterized by comparison of the physical data (mp, IR, and ^1^H and ^13^C NMR spectra) with that found in the literature [19, 20]. The structures of new 2-benzyl amino-1,3-benzoxazine compounds **15b–g** were confirmed using IR, ^1^H NMR, and ^13^C NMR spectroscopy and microanalysis. In the ^1^H spectra the CH_2_ of the benzyl amine appears as a doublet at ~*δ* 4.5 ppm. The previously analysed ^1^H and ^13^C NMR spectra of the parent 2-methylthio-1,3-benzoxazines **14** [35] were used to aid in the analysis of the new products **15b–g** s.

#### 2.1.6. Synthesis of 2-Oxo-substituted-benzoxazines **22a–h** and **25**

2-Methylthio-substituted-benzoxazines **14a**, **b**, and **d–g** were allowed to react with 10% HCl according to the general procedure E and gave 2-dione-1,3-benzoxazines **22a–h** with good to excellent yield (Scheme 5); however, product **22h** was produce if 40% HCl was used in the hydrolysis of **14h**. Similarly, the 2,8-dioxo product **25** was prepared from the hydrochloric acid hydrolysis of the corresponding 2,8-dimethylthio-analogue **24** (Scheme 5).

#### 2.1.7. Synthesis of Substituted-N-(benzyl carbamoyl)-2-hydroxybenzamides **23a–g**

The synthesis of the substituted-N-(benzyl carbamoyl)-2-hydroxybenzamides **23a–g** was achieved using the reaction of the relevant benzoxazine-2, 4-di-one **22a–h** with excess benzylamine in dioxane with refluxed according to the general procedure **F**.

*Structure Elucidation of Substituted-N-(benzyl carbamoyl)-2-hydroxybenzamides* ***23a–g***. The structures of the newly prepared substituted urea compounds **23a–g** were confirmed using IR and ^1^H and ^13^C NMR spectroscopy and microanalysis. The ^1^H NMR and IR spectra supported the proposed structures and showed some correlation with the previously prepared benzyl thiourea **13a–q**, **20a**, **c**, and **21**. In the ^1^H spectra, the methylene CH_2_ (H-11 of compounds **23a–g**) of the benzyl amine appears to shift up field as a doublet at ~*δ* 4.3 ppm. The NH (H-10 of compounds **23a–g**) appears as a broad triplet at ~*δ* 8.5 ppm. The ^1^H and ^13^C NMR spectra of the parent 2-oxo-1,3-benzoxazines **22** and **25**.

### 2.2. Biological Testing

#### 2.2.1. Broth Dilution Susceptibility Testing

In this study, some of the newly prepared compounds were tested and showed antimicrobial activity against 8 different bacterial strains and 4 cultures of fungi. The bacterial species investigated were *P. aeruginosa, B. subtilis, S. aureus, A. baumannii, E. coli, S. agalactiae M. smegmatis*, and *M. chlorophenolicum*. The antifungal evaluation was determined against *A. niger*, *A. corymbifera*, *R. oryzae,* and *A. alternata*. The minimal inhibitory concentrations (MICs) and minimal fungicidal concentrations (MFCs), defined as the lowest concentration of drug that inhibits the growth of bacteria or fungi in the inoculums', were determined using the broth dilution methods. The compounds which demonstrated MIC and MFC values less than 300 and 200 *μ*g mL^−1^, respectively, are listed in Tables 1 and 2. According to [36], antimicrobial agents are effective on a range of bacterial species at low concentrations, that is, <128 *μ*g mL^−1^. Therefore, we conducted our MIC experiments using concentrations as high as 300 *μ*g mL^−1^. We also selected a range of bacterial and fungal species to test our newly synthesised compounds. Some of the species are potentially pathogenic to humans and animals and others are problematic in an environmental setting. Furthermore, the bacterial species selected have different cell wall compositions, that is, some are Gram-negative and some are Gram-positive strains. Some antimicrobial agents inhibit bacteria by interacting with components of the cell wall that are absent in Gram-negative bacteria [37]; therefore, the selection of strains was carefully selected with the possibility that an inhibitory compound would also hint to its mechanism of action. Based on the results obtained it is clear that the Gram-negative strains, that is, *P. aeruginosa*, *E. coli,* and *A. baumannii* were least affected by the compounds and when inhibition was observed it was at high levels 200 *μ*g mL^−1^ or higher. Interestingly, compound **9d** seemed to have a more dramatic effect Gram-positive strains with the exception of *M. smegmatis*. Despite the effects that some of the compounds had on the bacterial strains, it appears that these compounds are not so effective when tested on the four fungal cultures chosen with the exception of **9d** on *A. corymbifera*. 

Based on the results obtained for each of the newly synthesised compounds, it is evident that compound **9d** has more potent effect when compared to the others. This reveals that despite the fact that most of the compounds do not seem to have a noteworthy effect on the strains, compound **9d** is of interest and further investigation is required.

#### 2.2.2. Disc Diffusion Susceptibility Testing

Disc diffusion susceptibility testing was performed on compounds with poor solubility in broth dilution susceptibility testing. The preliminary antimicrobial testing was achieved using the standard agar disk diffusion methods. Compounds that inhibited certain bacteria or fungi are summarized in (Tables 3 and 4).

The concentrations of the prepared compounds were 10^−4^ *μ*g mL^−1^ (see Experimental). The control data is used to determine if the bacterial strains are resistant (R) or sensitive (S) to the prepared compounds tested. The disc diffusion assay was used as a preliminary guide for all compounds and used in correspondence with the broth dilution method for determining MIC/MFC values. This method is particularly useful when MIC/MFC values are unable to be determined using the broth dilution method due to the compounds insolubility. The insoluble compounds zones of inhibition therefore can be determined in millimeters relative to the control and used as a rough guide. Since the zone of inhibition of clearance may be affected by other parameters, such as, the nutrient agar depth of the plate and solvent used, the results shown using this method therefore should be used as a guide. MIC/MFC values are determined using the broth dilution method. All compounds that showed clearance zones are listed in Tables 3 and 4 and were tested in duplicate with the average given. Any zone of inhibition that was noted around the disc was considered sensitive and the zone of clearly was noted. These results are more useful for compounds that were difficult to dissolve, but equally, these results can indicate resistance if the compound does not diffuse through the agar properly. 

Based on the results obtained in Section 2.2.1, it is clear that compound **9d** is of interest. Based on the results obtained in Table 3, compound **9d** has an inhibitor effect on *M. smegmatis* but in the MIC study had no effect. This could indicate solubility problems with the compound when in solution; however, this is only speculative; further studies are required to reveal the cause. In addition, compound **9d** seems to have no inhibitory effect on *S. aureus* but in the MIC studies had a dramatic affect. This difference in result is unusual but clearly indicates that different methods could reveal different results and therefore it is important to perform both methods prior to further investigation on their inhibitory effects. 

In the MIC studies, we used 300 *μ*g mL^−1^ as the highest cut-off level. If a compound has an inhibitory effect on any strain that is greater than this level, then this should be revealed in the disc diffusion assay. However, further investigation is required as some of these compounds are dissolved in DMSO and when applied to bacterial cultures can come out of solution. The disc diffusion assays seem to indicate some sensitivity to fungal cultures despite the fact that they were undetectable in the MIC studies.

### 2.3. The Structure Activity Relationships of the Tested Compounds (Broth Dilution)

The results in Tables 1 and 2 show that the 4-(benzylamino)-2-hydroxybenzoic acid derivative **9d** showed the broadest range of activity of the compounds tested, exhibiting activity against the Gram-positive and Gram-negative bacteria and also *M. chlorophenolicum*. Furthermore, compound **9d** showed to be more active than others against *S. aureus* with an MIC value of 25 *μ*g/mL. Compounds **20a**,**c** and **21** (bis-thiourea products) were found to be particularly active towards Gram-positive *B. subtilis *at MIC values of 12.5, 25, and 25 *μ*g mL^−1^. In addition, compound **21** also showed activity towards *M. smegmatis *(MIC 50 *μ*g mL^−1^). Other synthesized compounds which showed an inhibitory effect were **13n** which had an inhibitory effect on four bacterial species at 300 *μ*g mL^−1^ and **13d** and **13m** which had an inhibitory effect on two of the bacterial species at concentration 200 *μ*g mL^−1^. Interestingly, *E. coli* was not inhibited by any of the compounds. 

### 2.4. The Chemical Compounds Activity and Structural Relationships of the Antimicrobial Assay Results (From Disk Diffusion Assay)

In the presence of a compound, a zone of clearing was greater than the control which was indicative that the strain was sensitive to the compound, whereas a zone of clearing equal to the control indicated resistance. The results reveal that none of the compounds had an inhibitory effect on *E. coli *at concentration 10^4^ *μ*g mL^−1^ (Table 3). The *B. subtilis* bacterial species tested showed inhibitory effects to most of the compounds tested, for example, **13d** inhibited *S. aureus* most strongly and compounds **13k**, **13f**, and **13d** inhibited growth of *A. baumannii, B. subtilis,* and* S. agalactiae *(Table 3). Some bacterial species that were sensitive to a compound showed similar sized zones of inhibition. One example was **13k** which exhibited activity of 2 mm for *A. baumannii* and *B. subtilis* and 5 mm for *S. aureus* and *S. agalactiae.* The same applies to compounds **8d**, **9c**, and **9d** which had shown a 2 mm clearance zone against *P. aeruginosa M. smegmatis M. chlorophenolicum. *Similarly to the broth dilution results, compounds **21** and **9d** were found to be active against three fungi species, *R. oryzae, A. niger,* and *A. corymbifera,* with clearance zones 2–5 mm, respectively.

All the compounds tested showed a 2–4 mm zone of clearing for most of the susceptible species with the exception of compound **13b** which had a larger 6 mm zone of inhibition. This larger zone indicates a hypersensitive effect on the bacterial species; however, it is specific for the compound and species. Because the mechanism of action of the compound is unknown, it is difficult to explain the reason for the hypersensitive effect. One possible explanation is that *B. subtilis *encodes a protein that can transport compound **13b** into the cell and this has a more toxic effect than those working from outside the cell. A similar phenomenon has been shown with bacterial mercury resistance where the presence of a mercury import protein displays a larger zone of clearing in a disc diffusion assay [38].

The data obtained revealed patterns of inhibition, especially those conducted with the disc diffusion assay. This suggests that a similar mechanism of action could be involved in the inhibition of growth.

## 3. Conclusion 

In conclusion, we have prepared seven new compounds of 2-benzylamino-substituted-1,3-benzoxazines, nineteen new N-(benzyl carbamothioyl)-substituted-benzamide and have evaluated some for their activity against bacteria and fungi. It appears that N-(benzyl carbamothioyl)-substituted-benzamide has shown antibacterial activity such as **20a**, **20c**, **21**, **13d**, **13m**, and **13n**.

We are in the process of synthesising new substituted products by replacing the benzyl group of N-(benzyl carbamothioyl) by 6-aminopenicillanic acid and test their bacteria activity.

## 4. Experimental

### 4.1. Chemistry

Infrared spectra were obtained using a Perkin Elmer FT-IR 1720x spectrometer. ^1^H NMR and ^13^C NMR spectra were obtained using a Bruker AC 200 NMR spectrometer at 200 and 50 MHz, respectively. All ^1^H NMR and ^13^C NMR spectral results are recorded as chemical shifts (*δ*) relative to the internal TMS for proton and 77.0 ppm in CDCl_3_ solvent and 39.4 ppm in DMSO-d_6_ solvent for ^13^C NMR. Microanalysis was performed by Chemical and Micro analytical Services (CMAS), Australia. Melting point determinations were carried out using a Stuart Scientific (SMP3) melting point apparatus and all melting points are uncorrected.

#### 4.1.1. Starting Materials

The stating reagents benzyl amine, sodium hydrogen carbonate, methyl iodide-amino-2-hydroxybenzoic acid, and dry 1,4-dioxane were purchased from Aldrich Chemical Company and were used as received.

#### 4.1.2. Synthesis of 4-(Acetyl amino)-2-hydroxybenzoic Acid **8a**

According to the previously reported method [29, 30, 37], product **8a** was prepared from the reaction of 4-amino-2-hydroxybenzoic acid **7** and acetic anhydride and recrystallised from 1,4-dioxane, 82% yield, mp 221–224°C (lit [37] and mp 235°C). The physical and spectroscopic data is consistent with the literature values [27, 35].

#### 4.1.3. Synthesis of 4-Substituted ((Benzylidene)amino)-2-hydroxybenzoic Acid Intermediates **8b–e**

According to the previously reported method [29, 30], intermediates **8b–e** were prepared from the appropriate substituted benzaldehyde and 4-amino-2-hydroxy-benzoic acid **7**.

Products **8b**, **c**, and **e** were not identified and used immediately in the synthesis of compound **9a**, **b**, and **d**.

*(Z)-4-((3-Ethoxy-2-hydroxybenzylidene)amino)-2-hydroxybenzoic Acid* ***8d***. 3-Ethoxy-2-hydroxybenzaldehyde (1.66 g, 0.01 mol) was allowed to react with 4-amino-2-hydroxybenzoic acid 7 (1.53 g, 0.01 mol) for 1 hour according to the reported procedure [29, 30] and gave solid which recrystallised from methanol to give **8d** 2.95 g, 98% as red crystals, mp 185–188°C decomp. *ν*_max_ (KBr)/cm^−1^ 1655 (C=O), 1622, 1600 (C=N): ^1^HNMR (200 MHz, 300 K, d_6_-acetone) *δ* 8.93 (s, 1H, H-8), 8.02 (d, 1H, *J*_H6,H5_ = 8.8 Hz, H-6), 7.33 (dd, 1H, *J*_H15,H14_ = 7.8 Hz, *J*_H15,H13_ = 1.6 Hz, H-15), 7.24 (dd, 1H, *J*_H13,H14_ = 8.0 Hz, *J*_H13,H15_ = 1.6 Hz, H-13), 6.91–7.09 (m, 3H, H-3,H-5, and H-14), 4.24 (q, 2H, J_H16,H17_ = 7.0 Hz, H-16), 1.41 (t, 3H, J_H17,H16_ = 7.0 Hz, H-17). Product **8d** was used immediately in the synthesis of **9d**.

#### 4.1.4. Synthesis of 4-Substituted-(benzylamino)-2-hydroxybenzoic Acids **9a–d**

*General Procedure A.* In slight modification to a previous reported method, [28] the appropriate 4-substituted ((benzylidene) amino)-hydroxybenzoic acids **8b-c** reduced using sodium borohydride (2 equiv). 

*4-(Benzylamino)-2-hydroxybenzoic Acid* ***9a***. 2-Hydroxy-4-{[(*E*)-benzylidene]  amino} benzoic acid **8b** (2.41 g, 10 mmol) was allowed to react with sodium borohydride (0.76 g, 20 mmol) according to general procedure A. The resulting solid was recrystallised from methanol/water to give **9a** (1.76 g, 73%), mp 122–125°C. *ν*_max_ (KBr)/cm^−1^ 3500–3200 br (OH), 3024, 2569 (NH), 1632(C=O); ^1^HNMR (200 MHz, 340 K d_6_-DMSO) *δ* 11.34 (bs, 1H, OH of COOH exchangeable with D_2_O), 7.5 (d, 1H, *J*_H6,H5_ = 8.6 Hz, H-6), 7.21–7.47 (m, 6H, 5 x CH, 8-NH exchangeable with D_2_O), 6.22 (dd, 1H, *J*_H5,H6_ = 8.6 Hz, *J*_H5,H8_ = 2.0 Hz, H-5), 6.03 (d, 1H, *J*_H3,H5_ = 2.0 Hz, H-3), 4.38 (s, 2H, H-9). 3.32 (2-OH under the water envelope); ^13^C NMR (50 MHz, 340 K, d_6_-DMSO) *δ* 171.7 (C-7), 163.2 (C-2), 154.8 (C-4), 139.1 (C-10), 130.1 (C-6), 128.1, 126.9, 126.6 (C-12, C-11 and C-13), 105.3 (C-5), 100.4 (C-1), 96.8 (C-3), 45.8 (C-9). The resulting product **9a** was not stable and was used immediately in the genral procedure B. 

*2-Hydroxy-4-((2-hydroxybenzyl)amino)benzoic Acid* ***9b***. (*E*)-2-Hydroxy-4-((2-hydroxybenzylidene)amino)benzoic acid **8c** (2.6 g, 10 mmol) was allowed to react with sodium borohydride (0.76 g, 20 mmol) according to general procedure A. The resulting solid was recrystallised from methanol/water to give **9b** as white crystals (2.20 g, 85%), mp 184–186°C decomp. *ν*_max_ (KBr)/cm^−1^ 3500–3200 br (OH), 1614 (C=O); ^1^HNMR (200 MHz, 300 K d_6_-DMSO) *δ* 11.45 (bs, 1H, 8-NH or 11-OH exchangeable with D_2_O), 9.61 (bs, 1H, 11-OH or 8-NH exchangeable with D_2_O) 7.44 (d, 1H, *J*_H6,H5_ = 8.8 Hz, H-6), 7.1–6.7 (m, 6H, H-13, H-15, H-14, H-12 and 11-OH), 6.28 (dd, 1H, *J*_H5,H6_ = 8.8 Hz, *J*_H5,H3_ = 1.1 Hz, H-5), 5.92 (d, 1H, *J*_H3,H5_ = 1.1 Hz, H-3); ^13^C NMR (50 MHz, 350 K, d_6_-DMSO) *δ* 171.9 (C-7), 163.5 (C-2), 155.2/155.0 (C-11/C-4), 131.2 (C-6), 128.5 (C-13), 127.9 (C-14), 125.0 (C-10), 119.1 (C-15), 115.3 (C-12), 105.6 (C-5), 100.6 (C-1), 97.0 (C-3), 41.1 (C-8). The resulting product **9b** was used immediately in the general procedure B. 

*4-((3-Ethoxy-2-hydroxybenzyl)amino)-2-hydroxybenzoic Acid* ***9c***. (*E*)-4-((3-Ethoxy-2-hydroxybenzylidene)amino)-2-hydroxybenzoic acid **8d** (10 mmol, 3.0 g) was allowed to react with sodium borohydride (20 mmol, 0.76 g) according to general procedure A. The resulting solid was collected and recrystallised from methanol/water to give **9d** (2.66 g, 81%) as white crystals, mp 159–161°C decomp. *ν*_max_ (KBr)/cm^−1^ 3500–3200 br (OH, NH absorption under the OH envelope), 1625 (C=O); ^1^HNMR (200 MHz, 300 K d_6_-DMSO) *δ* 7.38 (d, 1H, *J*_H6,H5_ = 8.6 Hz, H-6), 6.85–6.65 (m, 3H, H-13, H-14 and H-15), 6.35 (bs, 4H, 3 x OH and NH), 6.17 (dd, 1H, *J*_H5,H6_ = 8.6 Hz, *J*_H5,H3_ = 1.8 Hz, H-5), 5.87 (d, 1H, *J*_H3,H5_ = 1.8 Hz, H-3), 4.23 (s, 2H, CH_2_NH, H-9), 4.05 (q, 2H, *J*_H16,H17_ = 6.8 Hz, H-16), 1.35 (t, 3H, *J*_H17,H16_ = 6.8 Hz, H-17); ^13^C NMR (50 MHz, 300 K, d_6_-DMSO) *δ* 172.2 (C-7), 163.6 (C-2), 155.3 (C-4), 146.6 (C-12), 144.1 (C-11), 131.4 (C-6), 125.7 (C-10), 120.3 (C-14), 119.2 (C-15), 111.8 (C-13), 105.8 (C-1), 100.2 (C-5), 96.7 (C-3), 64.4 (C-16), 40.8 (C-9), 15.0 (C-17); Anal. Calcd. For C_16_H_17_NO_5_: C, 63.36; H, 4.62; N, 5.65. Found: C, 63.51; H, 4.48; N, 5.66.

#### 4.1.5. Synthesis of Substituted-dihydroxy-di-carboxylic Acids **16** and **17**

According to the previously reported general procedures [23–26], the appropriate substituted phenol was used in the synthesis substituted-dihydroxy-di-carboxylic acids **16** and **17**.

#### 4.1.6. Synthesis of 7-N-Substituted-amino-1,3-oxazines **10a**, **b**, and **11a**, **b**, and **d**

*General Procedure B*. The substituted-2-hydroxy benzoic acid was allowed to react with the freshly prepared Ph_3_P(SCN)_2_ according to previously reported general procedure [22, 34].

*N-(4-Oxo-2-thioxo-3,4-dihydro-2H-benz[e][1,3]oxazin-7-yl)acetamide* ***10a***. Slightly modified to the previously reported general procedure B [22, 34], 4-(acetyl amino)-2-hydroxybenzoic acid **8a** (1.56 g, 8 mmol) was allowed to react with freshly prepared Ph_3_P(SCN)_2_ (10 mmol) at room temperature for 2 hours then under reflux for 16 hours. At the completion of the reaction, the PbBr_2_ filter cake was washed by acetic acid (150 mL) to extract the desired product. The acetic acid filtrate was evaporated and minimal toluene was added to dissolve any oil with the product. The crude solid was filtered and recrystallised from ethanol to give **10a** (1.22 g, 65%) as light red crystals, mp 285–287°C decomp. *ν*_max_ (KBr)/cm^−1^ 3290, 3183 (9-NH), 3072, 2923 (3-NH), 1704 (C=O), 1188 (C=S); ^1^HNMR (200 MHz, 390 K, d_6_-DMSO) *δ* 13.38 (bs, 1H, 9-NH), 10.56 (s, 1H, 3-NH), 7.86 (d, 1H, *J*_H5,H6_ = 8.6 Hz, H-5), 7.77 (d, 1H, *J*_H8,H6_ = 1.8 Hz, H-8), 7.44 (dd, 1H, *J*_H6,H5_ *=* 8.6 Hz, *J*_H6,H8_ = 1.8 Hz, H-6), 2.11 (s, 3H, 11-CH_3_); ^13^C NMR (50 MHz, 330 K, d_6_-DMSO) *δ* 181.9 (C-2), 169.2 (C-10), 156.7 (C-8a), 146.0 (C-7), 127.3 (C-5), 116.3 (C-6), 109.6 (C-4a), 104.2 (C-8), 24.0 (C-11) 155.9 (C-4); Anal. Calcd. For C_10_H_8_N_2_O_3_S: C, 50.84; H, 3.41; N, 11.86 C. Found: C, 50.69; H, 3.53; N, 11.86.

*Synthesis of 7-Amino-2-thioxo-2H-benz[e][1,3]oxazin-4(3H)-one* ***10b***. A suspension of (*E*)-4-((3-ethoxy-2-hydroxybenzylidene)amino)-2-hydroxybenzoic acid **8d** (1.2 g, 4 mmol) in dry DCM (20 mL) was added to a mixture of freshly prepared Ph_3_P(SCN)_2_ (10 mmol) according to the general procedure B. The resulting solid was isolated upon evaporation of the DCM filtrate **10b** (0.74 g, 91% crude yield). The solid was recrystallised from methanol, mp 250–253°C decomp. *ν*_max_ (KBr) cm^−1^ 3443, 3328, (NH_2_), 3059, 2916 (NH), 1749 (C=O), 1679, 1616 (C=C), 1205 (C=S); ^1^HNMR (200 MHz, 370 K, d_6_-DMSO) *δ* 13.04 (s, 1H, 3-NH), 7.58 (d, 1H, *J*_H5,H6_ = 8.6 Hz, H-5), 6.65 (s, 2H, 7-NH_2_), 6.60 (dd, 1H, *J*_H6,H5_ = 8.6 Hz, *J*_H6,H8_ =1.8 Hz, H-6), 6.37 (d, 1H, *J*_H8,H6_ = 1.8 Hz); ^13^C NMR (50 MHz, 330 K, d_6_-DMSO) *δ* 182.4 (C-2), 157.3, 156.9, 156.5 (C-4, C-8a, C-7), 128.1 (C-5), 112.7 (C-6), 102.3 (C-4a), 96.8 (C-8); Anal. Calcd. For C_8_H_6_N_2_O_2_S: C, 49.47; H, 3.11; N, 14.42. Found: C, 49.51; H, 3.20; N, 14.36.

*7-(Benzylamino)-2-thioxo-2H-benz[e][1,3]oxazin-4(3H)-one* ***11a***. In slight modification to previously reported general procedure B, 4-(benzylamino)-2-hydroxybenzoic acid **9a** (0.94 g, 4 mmol) was allowed to react with the freshly prepared Ph_3_P(SCN)_2_ (10 mmol) at room temperature for 2 hours then under reflux for 16 hours. The resulting crude solids (0.94 g, 82%) were filtered, collected, and recrystallised from toluene to give **11a** (0.88 g, 77%) as yellow crystals, mp 210–212°C decomp. *ν*_max_ (KBr)/cm^−1^ 3301 (9-NH) 3068, 2926 (3-NH), 1689 (C=O), 1197 (C=S); ^1^HNMR (200 MHz, 340 K, d_6_-DMSO) *δ* 12.90 (bs, 1H, 3-NH exchangeable with D_2_O), 7.61 (d, 1H, *J*_H5,H6_ = 8.8 Hz, H-5), 7.37–7.26 (m, 6H, Ar and 9-NH exchangeable with D_2_O), 6.73 (dd, 1H, *J*_H6,H5_ = 8.8 Hz, *J*_H6,H8_ = 2.0 Hz, H-6), 6.42 (d, 1H, *J*_H8,H6_ = 2.0 Hz, H-8), 4.41 (d, 2H, *J*_H10,H9_ = 5.9 Hz, H-10); ^13^C NMR (50 MHz, 340 K, d_6_-DMSO) *δ* 182.1 (C-2), 157.2, 156.5 (C-4, C-8a), 155.2 (C-7), 138.2 (C-11), (128.2, 127.3, 127.0, 126.8), (C-13, C-12, C-14 and C-5), 111.9 (C-6), 102.5 (C-4a), 95.1 (C-8), 45.8 (C-10); Anal. Calcd. For C_15_H_12_N_2_O_2_S: C, 59.99; H, 4.03; N, 9.33. Found: C, 59.83; H, 4.14; N, 9.45. 

*7-((2-Hydroxybenzyl)amino)-2-thioxo-2H-benz[e][1,3]oxazin-4(3H)-one* ***11b***. In slight modification to previously reported general procedure B, 2-hydroxy-4-((2-hydroxybenzyl)amino)benzoic acid **9b** (1.07 g, 4 mmol) was allowed to react with the freshly prepared Ph_3_P(SCN)_2_ (10 mmol) at room temperature for 2 hours then under reflux for 16 hours. The resulting crude solid recrystallised from acetonitrile to give **11b** (0.55 g, 46%) as yellow crystals, mp 173–175°C decomp. *ν*_max_ (KBr)/cm^−1^ 3500–3000 br (OH), 3310 (9-NH), 2926 (3-NH), 1684 (C=O), 1192 (C=S); ^1^HNMR (200 MHz, 340 K, d_6_-DMSO) *δ* 12.86 (bs, 1H, 3-NH), 9.50 (bs, 1H, 12-OH), 7.59 (d, 1H, *J*_H5,H6_ = 8.6 Hz, H-5), 7.41 (bs, 1H, 9-NH), 7.05–7.20 (m, 2H, H-14, H-16), 6.69–6.88 (m, 3H, H-13, H-15 and H-6), 6.42 (d, 1H, *J*_H8,H6_ = 2.0 Hz, H-8), 4.32 (s, 2H, H-10); ^13^C NMR (50 MHz, 340 K, d_6_-DMSO) *δ* 182.2 (C-2), 157.3, 156.7, 155.4, 154.9 (C-4, C-8a, C-12 and C-7), 128.5, 127.9, 127.3 (C-14, C-16 and C-5) 123.9 (C-11), 118.8, 115.1 C-15, C-13), 111.9 (C-6), 102.2 (C-4a), 94.9 (C-8), 40.9 (C-10); Anal. Calcd. For C_15_H_12_N_2_O_3_: C, 59.99; H, 4.03; N, 9.33 Found: C, 59.83; H, 4.14; N, 9.45.

*7-((3-Ethoxy-2-hydroxybenzyl)amino)-2-thioxo-2H-benz[e][1,3]oxazin-4(3H)-one* ***11c***. In slight modification to previously reported general procedure [22, 34], 4-((3-ethoxy-2-hydroxybenzyl)amino)-2-hydroxybenzoic acid **9d** (1.21 g, 4 mmol) was allowed to react with the freshly prepared Ph_3_P(SCN)_2_ (10 mmol) heated to room temperature for 2 hours then under reflux for 16 hours. The resulting solids (1.94 g) were recrystallised from acetic acid/water to give **11c** (0.95 g, 68%) as yellow crystals, mp 227–229°C decomp. *ν*_max_ (KBr)/cm^−1^ 3500–3200 br (OH), 3496 (7-NH), 3301 (3-NH), 1686 (C=O), 1620 (C=C), 1196 (C=S); ^1^HNMR (200 MHz, 390 K, d_6_-DMSO) *δ* 12.86 (bs, 1H, 3-NH), 8.44 (s, 1H, 12-OH exchangeable with D_2_O), 7.59 (d, 1H, *J*_H5,H6_ = 8.4 Hz, H-5), 7.42 (t, 1H, 9-NH) 6.89–6.40 (m, 4H, Ar and H-6), 6.41 (d, 1H, *J*_H8,H6_ = 1.8 Hz, H-8), 4.33 (d, 2H, 10-CH_2_), 4.07 (q, 2H, *J*_H17,H18_ = 7.0 Hz, 17-CH_2_), 1.36 (t, 3H, *J*_H18,H17_ = 7.0 Hz, 18-CH_3_); ^13^C NMR (50 MHz, 390 K, d_6_-DMSO) *δ* 181.8 (C-2), 156.9, 156.1, 155.2 (C-4, C-8a, C-7), 146.2, 144.2 (C-13, C-12), 126.9, 124.4 (C-15, C-11), 120.2, 118.4 (C-5, C-16), 112.2, 111.5 (C-14, C-6), 102.1 (C-4a), 94.8 (C-8), 64.2 (C-17), 40.7 (C-10), 14.0 (C-18); Anal. Calcd. For C_15_H_12_N_2_O_2_S: C, 59.29; H, 4.68. Found: C, 59.07; H, 4.70.

#### 4.1.7. Synthesis of Dithioxo-benz-bis-(1,3-oxazine)-diones **18a–c** and **19**

*2,8-Dithioxo-2,3,7,8-tetrahydrobenzo[1,2-e: 5,4-e* ′ *]bis([1,3]oxazine)-4,6-dione* ***18a***. In slight modification to the general procedure B, 4,6-dihydroxyisophthalic acid **16a** (0.79 g, 4 mmol) was allowed to react with the freshly prepared Ph_3_P(SCN)_2_ (10 mmol) heated to room temperature for 3 hours then under reflux for 5 hours. At the completion of the reaction, the reaction mixture was filtered and the PbBr_2_ filter cake washed with THF (100 mL) to extract product **18a**. Both THF and DCM filtrates were evaporated to dryness and minimal toluene added to remove any oil which may be present. The crude solid was recrystallised using dioxane/chloroform to give **18a** (0.63 g, 56%) as yellow crystals, mp >300°C decomp. *ν*_max_ (KBr)/cm^−1^ 3104, 3031, 2939, 2856 (3 and 7-NH), 1698 (C=O), 1152 (C=S); ^1^HNMR (200 MHz, 350 K d_6_-DMSO) *δ* 13.65 (bs, 2H, 2 x NH), 8.27 (bs, 1H, H-5), 7.69 (bs, 1H, H-10); ^13^C NMR (50 MHz, d_6_-DMSO) *δ* 181.1 (C-2,8), 159.0 (C-4,6), 156.1 (C-9a,10a), 126.5 (C-5), 113.8 (C-4a,5a), 104.1 (C-10); Anal. Calcd. For C_10_H_4_N_4_O_4_S_2_: C, 42.85; H, 1.44; N, 9.99. Found: C, 42.71; H, 1.48; N, 10.05.

*10-Hydroxy-2,8-dithioxo-2,3,7,8-tetrahydrobenzo[1,2-e: 5,4-e* ′ *]bis([ 1,3]oxazine)-4,6-dione* ***18b***. In slight modification to general procedure B, 4,5,6-trihydroxyisophthalic acid **16b** (0.86 g, 4 mmol) was allowed to react with the freshly prepared Ph_3_P(SCN)_2_ (10 mmol) at room temperature for 3 hours and then under reflux for 5 hours. At the completion of the reaction, the mixture was filtered and the PbBr_2_ cake washed with 100 mL 1,4-dioxane. Both dioxane and DCM filtrates were evaporated to dryness under reduced pressure and minimal toluene was added to remove any oil which may be present. The resulting solid was recrystallised from 1, 4 dioxane/chloroform to give **18b** (0.55 g, 46%) as yellow crystals, mp 286–289°C decomp. *ν*_max_ (KBr)/cm^−1^ 3300–3000 br (OH), 3103, 3047, 2938 (NH), 1698 (C=O), 1227 (C=S); ^1^H NMR (200 MHz, 390 K d_6_-DMSO) *δ* 13.69 (bs, 2H, 2 x NH), 11.64 (bs, 1H, OH), 7.80 (bs, 1H, H-5); ^13^CNMR (50 MHz, 390 K d_6_-DMSO) *δ* 181.0 (C-2,8), 156.8 (C-4,6), 148.6 (C-9a,10a), 131.9 (C-10), 113.8 (C-5), 113.8 (C-4a,5a); Anal. Calcd. For C_10_H_4_N_4_O_4_S_2_: C, 42.85; H, 1.44; N, 9.99. Found: C, 42.71; H,1.48; N, 10.05.

*10-Methyl-2, 8-dithioxo-2, 3, 7, 8-tetrahydrobenzo[1,2-e: 5,4-e* ′ *]bis([1*,*3*]*oxazine)-4,6-dione **18c***. In slight modification to general procedure B, 4,6-dihydroxy-5-methylisophthalic acid **16c** (1.7 g, 8 mmol) was allowed to react with freshly prepared Ph_3_P(SCN)_2_ [22, 34] (10 mmol) at room temperature for 3 hours then under reflux for 5 hours. At the completion of the reaction, the reaction mixture was filtered and the PbBr_2_ cake washed with approx 100 mL THF and filtered. Both THF and DCM filtrates were evaporated to dryness under reduced pressure and minimal toluene was added to remove any oil, which may be present. The solid which remained was then recrystallised using THF to give product **18c** (1.18 g, 50%). The physical and spectroscopic data is consistent with the literature values [22].

*2,9-Dithioxo-2,3,8,9-tetrahydrobenzo[1,2-e:4,3-e* ′ *]bis([1,3]oxazine)-4,7-dione* ***19***. In slight modification to the general procedure B [22, 34], 2,3-dihydroxyterephthalic acid **17** (0.86 g, 4 mmol) was allowed to react with freshly prepared Ph_3_P(SCN)_2_ (10 mmol) at room temperature for 3 hours then under reflux for 5 hours. At the completion of the reaction, the reaction mixture was filtered and the PbBr_2_ cake was washed with THF (100 mL). Both THF and DCM filtrates were evaporated to dryness under reduced pressure and minimal toluene was added to remove any oil, which may be present. The remaining solid is recrystallised from ethyl acetate to give **19** (0.54 g, 50%) as yellow crystals, mp > 300°C decomp. *ν*_max_ (KBr)/cm^−1^ 3079, 2904, 2864 (NH), 1718 (C=O), 1252 (C=S); ^1^H NMR (200 MHz, 390 K d_6_-DMSO) *δ* 13.90 (bs, 2H, 2 x NH), 7.89 (s, 2H, H-5 & H-6); ^13^C NMR (50 MHz, 390 K (d_6_-DMSO) *δ* 181.1 (C-2,9), 157.0 (C-4,7), 142.7 (C-10a,10b), 122.5 (C-5,6), 121.3 (C-4a,6a); Anal. Calcd. For C_10_H_4_N_2_O_4_S_2_: C, 42.85; H, 1.44; N, 9.99. Found: C, 42.74; H, 1.50; N, 9.93.

#### 4.1.8. Synthesis of Benzyl Thiourea **13a–q**

*General Procedure C.* The appropriate 2-thio-1,3-benzoxazines (1.7 mmole) **6**, **10a**,**b**, **11c**, and **12a–m** were suspended in a mixture of sodium bicarbonate (1 gm) and water (5 mL)/methanol (5 mL) with stirring, then the reaction mixture was warm to 40°C for few minutes then benzyl amine (4.25 mmol) was added dropwise, directly from the a pipette, left stirring at room temperature for 4 hours. At the completion of the reaction, the mixture was evaporated to dryness under reduced pressure and the pH was adjusted to 5-6 by using conc. HCl. The resulting solid was collected by vacuum filtration and washed with minimal water and recrystallized from an appropriate solvent.

*N-(Benzyl carbamothioyl)-2-hydroxybenzamide* ***13a***. 2-Thioxo-2*H*-benz[*e*]-1,3-oxazin-4(3*H*)-one **12a** was allowed to react with benzyl amine according to general procedure C. The crude solid was collected and recrystallized from toluene to give **13a** (75% yield), mp 176-177°C (Lit. [19, 20] 179°C). *ν*_max_ (KBr)/cm^−1^ 3301 (N–H), 3200–2700 (O–H), 1661 (C=O), 1605 (C=S); ^1^H NMR (d_6_-DMSO) *δ* 12.42 (bs, 1H, H-2′), 11.41 (s, 1H, O–H), 11.00 (t, 1H, *J* = 5.4 Hz, H-4′), 7.70–7.00 (m, 9H, ArH), 4.9 (d, 2H, *J* = 5.2 Hz, H-5′); ^13^C NMR (d_6_-DMSO) *δ* 179.6 (C-3′), 168.3 (C-1′), 160.7 (C-2), 136.3 (C-6′), 135.9 (C-4), 129.0 (C-6), 128.1 (C-7′), 127.9 (C-8′), 127.5 (C-9′), 120.1 (C-5), 118.8 (C-3), 113.3 (C-1), 50.0 (C-5′).

*N-(Benzyl carbamothioyl)-2-hydroxy-3-methylbenzamide* ***13b***. 8-Methyl-2-thioxo-2*H*-benz[*e*]-1,3-oxazin-4(3*H*)-one **12b** was allowed to react with benzyl amine according to general procedure C. The crude solid was collected and recrystallized from toluene to give **13b** (79% yield), mp 220–223°C. *ν*_max_ (KBr)/cm^−1^ 3062, 2883 (N–H), 1696 (C=O), 1610 (C=S); ^1^H NMR (d_6_-DMSO) *δ* 11.32 (bs, 1H, H-2′), 10.73 (s, 1H, O–H), 8.80 (t, 1H, *J* = 5.4 Hz, H-4′), 7.82 (d, 1H, *J* = 7.5 Hz, H-6), 7.40–7.20 (m, ArH, H-4), 6.8 (t, 1H, *J* = 7.5 Hz, H-5), 4.43 (d, 2H, *J* = 6.0 Hz, H-5′), 2.20 (s, 3H, 8-CH_3_); ^13^C NMR (d_6_-DMSO) *δ* 169.9 (C-3′), 157.7 (C-1′), 152.9 (C-2), 137.5 (C-6′), 136.0 (C-4), 128.4 (C-7′), 127.3 (C-8′), 127.0 (C-9′), 126.9 (C-3), 126.5 (C-6), 119.0 (C-5), 115.1 (C-1), 42.9 (C-5′), 15.9 (CH_3_); Anal. Calcd. For C_16_H_16_N_2_O_3_: C, 67.59; H, 5.67; N, 9.85. Found: C, 67.10; H, 5.38; N, 9.25.

*N-(Benzyl carbamothioyl)-2-hydroxy-[1,1* ′ *-biphenyl]-3-carboxamide* ***13c***. 8-Phenyl-2-thioxo-2*H*-benz[*e*]-1,3-oxazin-4(3*H*)-one **12c** was allowed to react with benzyl amine according to general procedure C. The crude solid was collected and recrystallized from toluene to give **13c** (80% yield), mp 250–253°C. *ν*_max_ (KBr)/cm^−1^ 3062, 2883 (N–H), 1686 (C=O), 1595 (C=S); ^1^H NMR (d_6_-DMSO) *δ* 11.30 (s, 1H, 2′-N–H), 10.70 (s, 1H, O–H), 8.80 (t, 1H, *J* = 5.4 Hz, 4′-N–H), 7.90 (d, 1H, *J* = 7.6 Hz, H-6), 7.80 (d, 1H, *J* = 7.6 Hz, H-4), 7.60 (dd, 1H, *J*_H8,H10_ = 1.6 Hz, *J*_H8,H9_ = 8.0 Hz, H-8), 7.50 (m, 4H, H-5/H-9/H-10), 7.30–7.20 (m, ArH, H-4), 4.90 (d, 2H, *J* = 6.0 Hz, H-5′); ^13^C NMR (d_6_-DMSO) *δ* 169.4 (C-3′), 157.7 (C-1′), 155.3 (C-2), 137.2 (C-6′), 134.2 (C-4), 129.5 (C-6), 128.4 (C-7′), 127.3 (C-8′), 127.0 (C-9′), 126.3 (C-3), 120.1 (C-1), 118.9 (C-5), 50.1 (C-5′); Anal. Calcd. For C_15_H_14_N_2_O_2_S: C, 62.92; H, 4.93; N, 9.78. Found: C, 63.03; H, 4.88; N, 9.80.

*N-(Benzyl carbamothioyl)-5-bromo-2-hydroxybenzamide* ***13d***. 6-Bromo-2-thioxo-2*H*-benz[*e*]-1,3-oxazin-4(3*H*)-one **12d** was allowed to react with benzyl amine according to general procedure C. The crude solid was collected and recrystallized from toluene to give **13d** (82% yield), mp 185°C. *ν*_max_ (KBr)/cm^−1^ 3296, 3073 (N–H), 1678 (C=O), 1601 (C=S); ^1^H NMR (d_6_-DMSO) *δ* 12.42 (bs, 1H, H-2′), 11.41 (s, 1H, O–H), 11.00 (t, 1H, *J* = 5.4 Hz, H-4′), 7.90 (d, 1H, *J* = 2.2 Hz, H-6), 7.60 (dd, 1H, *J*_H4,H6_ = 2.2 Hz, *J*_H4,H3_ = 7.5 Hz, H-4), 7.40–7.30 (m, 5H, ArH), 7.00 (d, 1H, *J* = 7.5 Hz, H-3), 4.90 (d, 2H, *J* = 5.5 Hz, H-5′); ^13^C NMR (d_6_-DMSO) *δ* 179.4 (C-3′), 163.3 (C-1′), 155.9 (C-2), 137.4 (C-4), 137.3 (C-6′), 132.9 (C-6), 128.5 (C-7′), 127.6 (C-8′), 127.3 (C-9′), 119.7 (C-1), 118.8 (C-3), 111.1 (C-5), 48.3 (C-5′); Anal. Calcd. For C_15_H_13_BrN_2_O_2_S: C, 49.33; H, 3.59; N, 7.67. Found: C, 49.39; H, 3.68; N, 7.93.

*N-(Benzyl carbamothioyl)-5-ethoxy-2-hydroxybenzamide* ***13e***. 6-Ethoxy-2-thioxo-2*H*-benz[*e*]-1,3-oxazin-4(3*H*)-one **12e** was allowed to react with benzyl amine according to general procedure C. The crude solid was collected and recrystallized from toluene to give **13e** (87% yield), mp 210°C. *ν*_max_ (KBr)/cm^−1^ 3300, 3051 (N–H), 1665 (C=O), 1630 (C=S); ^1^H NMR (d_6_-DMSO) *δ* 12.00 (bs, 1H, H-2′), 11.32 (s, 1H, O–H), 11.12 (t, 1H, *J* = 5.4 Hz, H-4′), 7.50 (s, 1H, H-6), 7.30–7.10 (m, 7H, ArH/H-3/H-4), 4.80 (d, 2H, *J* = 5.5 Hz, H-5′), 4.10–4.00 (q, 2H, *J* = 6.7 Hz, O–CH_2_), 1.30–1.20 (t, 3H, *J* = 6.7 Hz, CH_3_); ^13^C NMR (d_6_-DMSO) *δ* 179.8 (C-3′), 164.3 (C-1′), 164.0 (C-2), 158.3 (C-5), 137.4 (C-6′), 128.5 (C-7′), 127.6 (C-8′), 127.3 (C-9′), 120.2 (C-1), 107.9 (C-3), 107.7 (C-4), 101.7 (C-6), 63.6 (O–CH_2_), 48.2 (C-5′), 14.5 (CH_3_); Anal. Calcd. For C_17_H_18_N_2_O_3_S: C, 61.80; H, 5.49; N, 8.48. Found: C, 61.90; H, 5.55; N, 8.61.

*N-(Benzyl carbamothioyl)-4-ethoxy-2-hydroxybenzamide* ***13f***. 7-Ethoxy-2-thioxo-2*H*-benz[*e*]-1,3-oxazin-4(3*H*)-one **12f** was allowed to react with benzyl amine according to general procedure C. The crude solid was collected and recrystallized from ethyl acetate to give **13f** (73% yield), mp 215–218°C. *ν*_max_ (KBr)/cm^−1^ 3307, 3071 (N–H), 1655 (C=O), 1506 (C=S); ^1^H NMR (d_6_-DMSO) *δ* 11.91 (bs, 1H, H-2′), 11.00 (s, 1H, O–H), 10.90 (t, 1H, *J* = 5.6 Hz, H-4′), 7.80 (d, 1H, *J* = 8.9 Hz, H-6), 7.30–7.20 (m, 5H, ArH), 6.60 (d, 1H, *J* = 8.9 Hz, H-5), 6.40 (sd, 1H, *J* = 1.5 Hz, H-3), 4.80 (d, 2H, *J* = 5.5 Hz, H-5′), 4.10–4.00 (q, 2H, *J* = 6.7 Hz, O–CH_2_), 1.30–1.20 (t, 3H, *J* = 6.7 Hz, CH_3_); ^13^C NMR (d_6_-DMSO) *δ* 179.8 (C-3′), 164.3 (C-1′), 164.0 (C-4), 158.3 (C-2), 137.4 (C-6′), 130.9 (C-6), 128.5 (C-7′), 127.6 (C-8′), 127.3 (C-9′), 109.2 (C-5), 107.9 (C-1), 101.7 (C-3), 63.6 (O–CH_2_), 48.2 (C-5′), 14.5 (CH_3_); Anal. Calcd. For C_17_H_18_N_2_O_3_S C, 61.80; H, 5.49; N, 8.48. Found: C, 62.02; H, 5.55; N, 8.51.

*N-(Benzyl carbamothioyl)-3-ethoxy-2-hydroxybenzamide* ***13g***. 8-Ethoxy-2-thioxo-2*H*-benz[*e*]-1,3-oxazin-4(3*H*)-one **12g** was allowed to react with benzyl amine according to general procedure C. The crude solid was collected and recrystallized from toluene to give **13g** (82% yield), mp 188–190°C. *ν*_max_ (KBr)/cm^−1^ 3310, 3080 (N–H), 1656 (C=O), 1525 (C=S); ^1^H NMR (d_6_-DMSO) *δ* 11.80 (s, 1H, 2′-N–H), 11.60 (s, 1H, O–H), 11.00 (t, 1H, *J* = 5.4 Hz, 4′-N–H), 7.30–7.20 (m, 6H, ArH/H-5), 7.10 (d, 1H, *J* = 7.5 Hz, H-6), 6.90 (d, 1H, *J* = 7.5 Hz, H-4), 4.80 (d, 2H, *J* = 5.5 Hz, H-5′), 4.00–3.90 (q, 2H, *J* = 6.7 Hz, O–CH_2_), 1.30–1.20 (t, 3H, *J* = 6.7 Hz, CH_3_); ^13^C NMR (d_6_-DMSO) *δ* 179.5 (C-3′), 164.3 (C-1′), 151.8 (C-3), 150.5 (C-2), 137.3 (C-6′), 128.5 (C-7′), 127.6 (C-8′), 127.3 (C-9′), 123.0 (C-5), 118.5 (C-6), 116.5 (C-1), 114.2 (C-4), 63.5 (O–CH_2_), 48.3 (C-5′), 14.6 (CH_3_); Anal. Calcd. For C_17_H_18_N_2_O_3_S: C, 61.80; H, 5.49; N, 8.48. Found: C, 61.96; H, 5.61; N, 8.54.

*N-(Benzyl carbamothioyl)-2-hydroxy-5-methoxybenzamide* ***13h***. 6-Methoxy-2-thioxo-2*H*-benz[*e*]-1,3-oxazin-4(3*H*)-one **12h** was allowed to react with benzyl amine according to general procedure C. The crude solid was collected and recrystallized from ethyl acetate to give **13h** (69% yield), mp 140°C. *ν*_max_ (KBr)/cm^−1^ 3313, 3081 (N–H), 1667 (C=O), 1610 (C=C), 1516 (C=S); ^1^H NMR (d_6_-DMSO) *δ* 12.40 (s, 1H, 2′-N–H), 11.30 (s, 1H, O–H), 11.20 (t, 1H, *J* = 5.4 Hz, 4′-N–H), 7.50–7.30 (m, 8H, ArH/H-3/H-4/H-6), 3.80 (s, 3H, O–CH_3_); ^13^C NMR (d_6_-DMSO) *δ* 179.9 (C-3′), 165.4 (C-1′), 151.5 (C-5), 149.0 (C-2), 137.4 (C-6′), 128.5 (C-7′), 127.6 (C-8′), 127.3 (C-9′), 121.8 (C-3), 117.9 (C-4), 116.4 (C-1), 56.2 (O–CH_3_), 48.1 (C-5′); Anal. Calcd. For C_16_H_16_N_2_O_3_S: C, 60.74; H, 5.10; N, 8.85. Found: C, 60.82; H, 5.18; N, 8.93.

*N-(Benzyl carbamothioyl)-2-hydroxy-4-methoxybenzamide* ***13i***. 7-Methoxy-2-thioxo-2*H*-benz[*e*]-1,3-oxazin-4(3*H*)-one **12i** was allowed to react with benzyl amine according to general procedure C. The crude solid was collected and recrystallized from ethyl acetate to give **13i** (87% yield), mp 210°C. *ν*_max_ (KBr)/cm^−1^ 3313, 3081 (N–H), 1667 (C=O), 1610 (C=C), 1520 (C=S); ^1^H NMR (d_6_-DMSO) *δ* 12.40 (s, 1H, 2′-N–H), 11.30 (s, 1H, O–H), 11.20 (t, 1H, *J* = 5.4 Hz, 4′-N–H), 7.70 (d, 1H, *J* = 7.5 Hz, H-6), 7.50–7.30 (m, 5H, ArH), 6.70 (m, 2H, H-3/H-5), 3.80 (s, 3H, O–CH_3_); ^13^C NMR (d_6_-DMSO) *δ* 179.9 (C-3′), 165.4 (C-1′), 160.5 (C-4), 159.0 (C-2), 137.4 (C-6′), 128.5 (C-7′), 127.6 (C-8′), 127.3 (C-9′), 126.8 (C-6), 117.9 (C-5), 116.4 (C-1), 106.0 (C-3), 56.2 (O–CH_3_), 48.1 (C-5′); Anal. Calcd. For C_16_H_16_N_2_O_3_S: C, 60.74; H, 5.10; N, 8.85. Found: C, 60.72; H, 5.20; N, 8.90.

*N-(Benzyl carbamothioyl)-2-hydroxy-3-methoxybenzamide* ***13j***. 8-Methoxy-2-thioxo-2*H*-benz[*e*]-1,3-oxazin-4(3*H*)-one **12j** was allowed to react with benzyl amine according to general procedure C. The crude solid was collected and recrystallized from toluene to give **13j** (82% yield), mp 190–193°C. *ν*_max_ (KBr)/cm^−1^ 3313, 3081 (N–H), 1667 (C=O), 1610 (C=C), 1518 (C=S); ^1^H NMR (d_6_-DMSO) *δ* 12.40 (s, 1H, 2′N–H), 11.30 (s, 1H, O–H), 11.20 (t, 1H, *J* = 5.4 Hz, 4′-N–H), 7.50-7.20 (m, 8H, ArH/H-4/H-5/H-6), 3.80 (s, 3H, O–CH_3_); ^13^C NMR (d_6_-DMSO) *δ* 179.9 (C-3′), 165.4 (C-1′), 149.5 (C-3), 148.0 (C-2), 137.4 (C-6′), 128.5 (C-7′), 127.6 (C-8′), 127.3 (C-9′), 121.8 (C-6), 117.9 (C-5), 116.4 (C-1), 116.0 (C-4), 56.2 (O–CH_3_), 48.1 (C-5′); Anal. Calcd. For C_16_H_16_N_2_O_3_S: C, 60.74; H, 5.10; N, 8.85. Found: C, 60.82; H, 5.18; N, 8.93.

*N-(Benzyl carbamothioyl)-2-hydroxy-3,5-diiodobenzamide* ***13k***. 6,8-diiodo-2-thioxo-2*H*-benz[*e*]-1,3-oxazin-4(3*H*)-one **12k** was allowed to react with benzylamine according to general procedure C. The solid was collected and recrystallized from toluene to give **13k** (75% yield), mp 173–175°C. *ν*_max_ (KBr)/cm^−1^ 3264, 3025 (N–H), 1633 (C=O), 1576 (C=C), 1545 (C=S); ^1^H NMR (d_6_-DMSO) *δ* 11.90 (s, 1H, 2′-N–H), 10.90 (t, 1H, *J* = 5.4 Hz, 4′-N–H), 8.20 (d, 1H, *J* = 2.2 Hz, H-6), 8.00 (d, 1H, *J* = 2.2 Hz, H-4), 7.40–7.30 (m, 5H, ArH), 4.90 (d, 2H, *J* = 5.5 Hz, H-5′), ^13^C NMR (d_6_-DMSO) *δ* 179.8 (C-3′), 165.6 (C-1′), 157.0 (C-2), 149.7 (C-4), 137.9 (C-6′), 137.3 (C-6), 128.5 (C-7′), 127.7 (C-8′), 127.3 (C-9′), 121.1 (C-1), 91.75 (C-5), 81.9 (C-3), 48.2 (C-5′); Anal. Calcd. For C_15_H_12_I_2_N_2_O_2_S: C, 33.48; H, 2.25; N, 5.21. Found: C, 33.43; H, 2.66; N, 5.78.

*N-(Benzyl carbamothioyl)-2,4-dihydroxybenzamide* ***13l***. 7-Hydroxy-2-thioxo-2*H*-benz[*e*]-1,3-oxazin-4(3*H*)-one **12l** was allowed to react with benzylamine according to general procedure C. The solid was collected and recrystallized from toluene to give **13l** (87% yield), mp190°C. *ν*_max_ (KBr)/cm^−1^ 3200–2700 (O–H), 3118, 3033 (N–H), 1665 (C=O), 1626 (C=C), 1554 (C=S); ^1^H NMR (d_6_-DMSO) *δ* 12.10 (bs, 1H, O–H), 11.30 (s, 1H, N–H), 11.20 (t, 1H, *J* = 5.4 Hz, N–H), 10.50 (O–H), 7.80 (d, 1H, *J* = 7.6 Hz, H-6), 7.30 (m, 5H, ArH), 6.40 (m, 2H, H-3/H-5), 4.80 (d, 2H, *J *= 5.7 Hz, H-5′); ^13^C NMR (d_6_-DMSO) *δ* 180.0 (C-3′), 164.7 (C-1′), 163.9 (C-4), 158.5 (C-2), 137.5 (C-6′), 133.3 (C-6), 128.7 (C-7′), 127.7 (C-8′), 127.5 (C-9′), 109.2 (C-5), 107.9 (C-1), 102.9 (C-3), 48.2 (C-5′); Anal. Calcd. For C_15_H_14_N_2_O_3_S: C, 59.59; H, 4,67; N, 9.27. Found: C, 59.88; H, 4.68; N, 9.59.

*N-(Benzyl carbamothioyl)-2,4-dihydroxy-3-methylbenzamide* ***13m***. 7-Hydroxy-8-methyl-2-thioxo-2*H*-benz[*e*]-1,3-oxazin-4(3*H*)-one **12m** was allowed to react with benzylamine according to general procedure C. The solid was collected and recrystallized from ethanol to give **13m** (78% yield), mp 193–195°C. *ν*_max_ (KBr) 3381, 3225 (O–H), 3020–2800 (N–H), 1639 (C=O), 1613 (C=S); ^1^H NMR (d_6_-DMSO) *δ* 11.60 (bs, 1H, 2-OH), 11.10 (t, 1H, *J* = 5.6 Hz, 4′-NH), 10.40 (bs, 1H, 2′-NH), 7.70 (d, 1H, *J*_H6,H5_ = 8.8 Hz; H-6), 7.40–7.30 (m, 6H/H-7′/H-8′/H-9′/4-OH), 6.50 (d, 1H, *J*_H5,H6_ = 8.8 Hz; H-5), 4.90 (d, 2H, *J* = 5.6 Hz, H-5′), 2.00 (s, 3H, 3-CH_3_); ^13^C NMR (d_6_-DMSO) *δ* 179.9 (C-3′), 166.1 (C-1′), 161.5 (C-4), 157.2 (C-2), 137.3 (C-6′), 129.1 (C-7′), 128.4 (C-8′), 127.5 (C-9′), 127.2 (C-6, C-3), 111.8 (C-1), 108.3 (C-5), 48.1 (C-5′), 8.6 (3-CH_3_). Anal. Calcd. For C_16_H_16_N_2_O_3_S C, 60.74; H, 5.10; N, 8.85. Found: C, 60.60; H, 5.08; N, 8.80; %.

*N-(Benzyl carbamothioyl)-2,3-dihydroxybenzamide* ***13n***. 8-Hydroxy-2-thioxo-2*H*-benz[*e*]-1,3-oxazin-4(3*H*)-one **12n** was allowed to react with benzyl amine according to general procedure C. The crude solid was collected and recrystallized from ethanol to give **13n** (72% yield), mp 172°C. *ν*_max_ (KBr)/cm^−1^ 3411, 2942 br (O–H), 3118, 3033 (N–H), 1661 (C=O), 1626 (C=C), 1553 (C=S); ^1^H NMR (d_6_-DMSO) *δ* 11.61 (s, 1H, 2′-N–H), 11.22 (t, 1H, *J* = 5.6 Hz, 4′-N–H), 10.53 (O–H), 7.30 (m, 7H, ArH/H-6), 7.00 (d, 1H, *J* = 7.6 Hz, H-4), 6.70 (t, 1H, *J* = 7.9 Hz, H-5), 4.80 (d, 2H, *J* = 5.7 Hz, H-5′); ^13^C NMR (d_6_-DMSO) *δ* 179.7 (C-3′), 165.0 (C-1′), 146.6 (C-2), 146.3 (C-3), 137.3 (C-6′), 128.5 (C-7′), 127.6 (C-8′), 127.3 (C-9′), 120.5 (C-5), 119.4 (C-4), 119.2 (C-5), 116.8 (C-1), 48.2 (C-5′); Anal. Calcd. For C_15_H_14_N_2_O_3_S: C, 59.59; H, 4.67; N, 9.27. Found: C, 60.08; H, 5.06; N, 9.59.

*4-Amino-N-(benzyl carbamothioyl)-2-hydroxybenzamide* ***13o***. 7-Amino-2-thioxo-2*H*-benz[*e*][1,3]oxazin-4(3*H*)-one **10b** was allowed to react with benzylamine following general procedure C. The resulting solid was collected and recrystallised from methanol/water to give **13o** (0.35 g, 78%) as off white solid, mp 290–293°C decomp. *ν*_max_ (KBr)/cm^−1^ 3500–3200 (OH), 3442, 3332 (NH), 1750, 1677 (C=O), 1388 (C=S);. ^1^HNMR (200 MHz, 300 K, d_6_-DMSO) *δ* 11.54 (s, 1H, 8-NH exchangeable with D_2_O), 11.33–11.28 (bm, 2H, 10-NH, 2-OH exchangeable with D_2_O), 7.60 (d, 1H, *J*_H6,H5_ = 8.6 Hz, H-6), 7.33–7.29 (m, 7H, Ar, H-11, H-12, H-13 and 4-NH_2_ exchangeable with D_2_O), 6.21 (dd, 1H, *J*_H5,H6&H5H3_ = 8.6 Hz, *J*_H5,H3_ = 1.6 Hz, H-3), (d, 1H, *J*_H3,H5_ = 1.6 Hz, H-3), 4.84 (d, 2H, *J*_H11,H10_ = 5.7 Hz, H-11); ^13^C NMR (50 MHz, 300 K, d_6_-DMSO) *δ* 180.3 (C-9), 165.2 (C-7), 158.6 (C-2), 155.1 (C-4), 137.7 (C-12), 133.2 (C-6), 128.9 (C-14), 127.8, 127.7 (C-13, C-15), 108.2 (C-5), 104.3 (C-1), 99.7 (C-3), 48.4 (C-11); Anal. Calcd. For C_15_H_15_N_3_O_2_S: C, 59.78; H, 5.02; N, 13.94. Found: C, 59.55; H, 5.08; N, 13.72.

*4-Acetamido-N-(benzyl carbamothioyl)-2-hydroxybenzamide* ***13p***. *N*-(4-Oxo-2-thioxo-3, 4-dihydro-2*H*-benz[*e*][1,3]oxazin-7-yl) acetamide **10a** was allowed to react with benzylamine following general procedure C. The resulting solid was collected and recrystallised from ethanol to give **13p** (0.27 g, 47%) as off white crystals. mp 263–266°C. *ν*_max_ (KBr)/cm^−1^ 3500–3200 (OH), 3292, 3113, (NH), 1656 (C=O), 1374 (C=S); ^1^HNMR (200 MHz, 300 K, d_6_-DMSO) *δ* 12.09 (s, 1H, 16-NH), 11.40 (s, 1H, 8-NH), 11.18 (t, 1H, *J*_H10,H11_ = 4.6 Hz, 10-NH), 10.26 (s, 1H, 2-OH), 7.84 (d, 1H, *J*_H6,H5_ = 7.8 Hz H-6), 7.64 (s, 1H, H-3), 7.37–7.30 (m, 5H, Ar, H-13, H-14, H-15), 7.02 (d, 1H, *J*_H5,H6_ = 7.8 Hz, H-5), 4.85 (d, 2H, *J*_H11,H10_ = 4.8 Hz, H-11), 2.07 (s, 3H, CH_3_); ^13^C NMR (50 MHz, 300 K, d_6_-DMSO) *δ* 179.7 (C-9), 169.1 (C-7), 164.2 (C-17), 157.4 (C-2), 145.3 (C-4), 137.3 (C-12), 131.9 (C-6), 128.5, 127.6, 127.3 (C-13, C-14, C-15), 111.0, 110.8 (C-1, C-5), 105.9 (C-3), 48.1 (C-11), 24.2 (C-18); Anal. Calcd. For C_17_H_17_N_3_O_3_S: C, 59.46; H, 4.97; N, 12.24. Found: C, 59.20; H, 5.07; N, 12.02.

*N-(Benzyl carbamothioyl)-4-((3-ethoxy-2-hydroxybenzyl)amino)-2-hydroxybenzamide* ***13q***. 3-Ethoxy-2-hydroxy-1,3-benzoxazine **11c** was allowed to react with benzylamine according to the general procedure C. The resulting solid was collected and recrystallised from acetonitrile to give **13q** (0.59 g, 77%) as yellow crystals, mp 201–204°C. *ν*_max_ (KBr)/cm^−1^ 3500–3200 (OH), 3496, 3395, 3256 (NH), 1686, 1646 (C=O), 1342 (C=S); ^1^HNMR (200 MHz, 300 K, d_6_-DMSO) *δ* 11.26 (8-NH), 8.33 (m, 1H, 2-OH), 7.63 (d, 1H, *J*_H6,H5_ = 9.0 Hz, H-6), 7.66–7.28 (m, 5H, Ar H-13, H-14, H-15), 7.1 (t, 1H, *J*_H16,H17_ = 5.1 Hz, H-16), 6.92–6.66 (m, 5H, H-21, H-22, H-25, 10-NH and 19-OH exchangeable with D_2_O), 6.30 (dd, 1H, *J*_H5,H6&H5,H3_ = 9.0 Hz, *J*_H5,H3_ = 1.6 Hz, H-5), 6.14 (d, 1H, *J*_H3,H5_ = 1.6 Hz, H-3), 4.85 (d, 2H, *J*_H11,H10_ = 5.1 Hz, H-11), 4.25 (d, 2H, *J*_H17,H16_ = 5.3 Hz, H-17), 4.08–4.01 (m, 3H, *J*_H24,H25_ = 7.0 Hz, H-24 and 16-NH), 1.45 (t, 3H, *J*_H25,H24_ = 7.0 Hz, H-25); ^13^C NMR (50 MHz, 340 K, d_6_-DMSO) *δ* 180.1 (C-9), 164.8 (C-7), 158.2 (C-2), 154.7 (C-4), 146.3, 144.1 (C-20, C-19), 137.2 (C-12), 132.1 (C-6), 128.2, 127.3, 127.0 (C-14, C-13, C-15), 125.1 (C-18), 119.9, 118.5 (C-21, C-23), 111.8 (C-22), 106.1, 103.6 (C-1, C-5), 97.2 (C-3), 64.1 (C-24), 47.9 (C-17), 40.8 (C-11), 14.4 (C-25); Anal. Calcd. For C_24_H_25_N_3_O_4_S*·*H_2_O: C, 61.39; H, 5.80; N, 8.95. Found: C, 61.59; H, 5.57; N, 9.38.

#### 4.1.9. *N1,N3-*Bis(benzyl carbamothioyl)-4,6-dihydroxy-lisophthalamide **20a**

In slight modification to the general procedure C, 2, 8-dithioxo-2, 3, 7, 8-tetrahydrobenzo[1,2-*e: *5,4-*e*′]bis([1,3]oxazine)-4,6-dione **18a** (1 mmol, 0.28 g) was allowed to react with benzylamine (3 mmol. 0.32 g) and sodium hydrogen carbonate solution (1 g in 12 mL methanol and 2.4 mL water) for 16 hour. The resulting solid was collected and recrystallised from ethanol to give **20a** (0.31 g, 63%) as off white crystals.mp 276–279°C decomp. *ν*_max_ (KBr)/cm^−1^ 3313, (NH), 1672 (C=O), 1328 (C=S), ^1^HNMR (200 MHz, 340 K, d_6_-DMSO) *δ* 11.25 (bs, 2H, 2 x NH), 11.06 (t, 2H, *J*_H10,H11_ = 5.7 Hz, H-10,18), 8.60 (s, 1H, H-6), 7.38–7.29 (10H, 2 x Ar), 6.63 (s, 1H, H-3), 4.87 (d, 4H, *J*_H11,H10_ = 5.7 Hz, H-11,20), 3.3 (OH under the water envelope); ^13^C NMR (50 MHz, 340 K, d_6_-DMSO) *δ* 179.5 (C-9,17), 163.3 (C-7,15), 161.5 (C-2,4), 137.0, 136.6 (C-11, 20 and C-6), 128.2, 127.3, 127.0 (C-13, 22, C-12, 21 and C-14, 25), 110.3 (C-1,15), 103.7 (C-3), 48.0 (C-11,19); Anal. Calcd. For C_24_H_22_N_4_O_4_S_2_: C, 58.28; H, 4.48; N, 11.33. Found: C, 58.37; H, 4.67; N, 11.22.

#### 4.1.10. *N1,N3-*Bis(benzyl carbamothioyl)-4,6-dihydroxy-5-methylisophthalamide **20c**

In slight modification to general procedure C, 10-methyl-2,8-bis(methylthio)benzo[1,2-*e: *5,4-*e*′]bis([1,3]oxazine)-4,6-dione **18c** (1 mmol, 0.32 g) was allowed to react with benzylamine (3 mmol. 0.32 g), Sodium hydrogen carbonate solution (1 g in 12 mL methanol and 2.4 mL water) for 16 h. The resulting solid was collected and recrystallised from ethanol to give **20c** (0.25 g, 49%) as off white crystals. mp 227–229°C decomp. *ν*_max_ (KBr)/cm^−1^ 3500–3200 (OH), 3414, 3257 (NH), 1651 (C=O), 1321 (C=S); ^1^HNMR (200 MHz, 340 K, d_6_-DMSO) *δ* 11.51 (bs, 2H, 2 x NH), 10.96 (s, 2H, *J*_H10-H11_ = 5.7 Hz, H-10,19), 8.48 (s, 1H, H-6), 7.39–7.28 (m, 10H, 2 x Ar), 4.90 (d, 2H, *J*_H9,H8_ = 5.7 Hz, H-11, 20), 2.10 (s, 3H, H-3), 3.3 (OH under the water envelope); ^13^C NMR (50 MHz, 340 K, d_6_-DMSO) *δ* 179.6 (C-9, 18), 166.3 (C-7,16), 161.3 (C-2, 4), 136.9 (C-12, 21), 131.2 (C-4), 128.1 (C-14, 23), 127.3, 127.0 (C-13, 22 and C-15, 24), 113.2 (C-1,5), 108.9 (C-3), 48.1 (C-11, 20), 8.4 (C-1, 5); Anal. Calcd. For C_25_H_25_N_4_O_4_S*·*H_2_O: C, 57.02; H, 4.98; N, 10.64. Found: C, 57.32; H, 4.48; N, 10.85.

#### 4.1.11. *N1,N4-*Bis(benzyl carbamothioyl)-2,3-dihydroxyterephthalamide **21**

In slight modification to the general procedure C, 2,9-dithioxo-2,3,8,9-tetrahydrobenzo[1,2-*e:*4,3-*e*′]bis([1,3]oxazine)-4,7-dione **19** (1 mmol, 0.28 g) was allowed to react with benzylamine (3 mmol. 0.32 g), sodium hydrogen carbonate solution (1 g in 12 mL methanol and 2.4 mL water) for 16 hour. The resulting solid was collected and recrystallised using ethanol to give 21 (0.31 g, 63%) as off white crystals mp 195–198°C. *ν*_max_ (KBr)/cm^−1^ 3500–3200 (OH), 3401, 3248 (NH), 1671, 1649 (C=O), 1338 (C=S); ^1^HNMR (200 MHz, 300 K, d_6_-DMSO) *δ* 11.88 (bs, 2 x NH, H-8,17), 11.07 (t, 1H, *J*_H10,H11_ = 5.9 Hz, H-10,19), 8.56 (bs, 2H, 2,3-OH), 7.39–7.28 (m, 12H, 2 x Ar and 2 x CH, H-13, H-14, H-15 and H-5,6), 4.88 (d, 4H, *J*_H11,H10_ = 5.9 Hz, H-11,20); ^13^C NMR (50 MHz, 340 K, d_6_-DMSO) *δ* 179.6 (C-9,18), 164.9 (C-7,16), 147.9 (C-2,3), 136.9 (C-12, 21), 128.2 (C-14,23), 127.3 (C-13,22), 127.0 (C-15,24), 120.9 (C-1,4), 119.2 (C-5,6); Anal. Calcd. For C_24_H_22_N_4_O_4_S_2_ *·*H_2_O: C, 56.23; H, 4.72; N, 11.33. Found: C, 55.79; H, 4.78; N, 10.93.

#### 4.1.12. Synthesis *N-*(2-(Methylthio)-4-oxo-4H-benz[e][1,3]oxazin-7-yl)acetamide **14h**

*N*-(4-Oxo-2-thioxo-3,4-dihydro-2*H*-benz[*e*][1,3]oxazin-7-yl)acetamide **10a** (0.59 g, 2.5 mmol) was allowed to react with methyl iodide following the previously reported [35]. The resulting beige solid **14h** (0.61 g, 97%) is collected and used without further purification but can be crystallised from ethanol, mp 268–269°C *ν*_max_ (KBr)/cm^−1^ 3278, 3142, 3110 (NH), 3062 (CH Ar), 2929 (CH Aliphatic), 1760, 1709, 1671 (C=O), 1615 (C=N), 1554 (C=C); ^1^HNMR (200 MHz, 300 K d_6_-DMSO) *δ* 10.60 (s, 1H, 9-NH), 7.88 (b, 1H, H-8), 7.87 (d, 1H, *J*_H5,H6_ = 8.6 Hz, H-5), 7.45 (d, 1H, *J*_H6,H5_ = 8.6 Hz, H-6), 2.58 (s, 3H, 11-CH_3_), 2.12 (s, 3H, 3′-CH_3_); ^13^C NMR (50 MHz, 300 k, d_6_-DMSO) *δ* 172.8 (C-2), 169.6 (C-10), 162.1 (C-4), 155.8 (C-8a), 145.2 (C-7), 128.0 (C-5), 117.5 (C-6), 112.1 (C-4a), 104.4 (C-8); Anal. Calcd. For C_15_H_12_N_2_O_2_S: C, 59.99; H, 4.03; N, 9.33. Found: C, 59.83; H, 4.14; N, 9.45.

#### 4.1.13. Synthesis of 2-Benzyl amino-1,3-benzoxazines **15a–h**

*General Procedure D.* The appropriate 2-methylthio-1,3-benzoxazine **14a–h** (2.5 mmol) was suspended in dry 1,4-dioxane (10 mL) in a 50 mL round-bottomed flask. Benzyl amine (12.5 mmol) was then added dropwise, directly from the pipette, with stirring, and then the reaction mixture was heated to reflux for 4 hours. At the completion of the reaction, the reaction mixture was evaporated to dryness under reduced pressure and triturated with minimal diethyl ether. The resulting solid product **14** was collected by vacuum filtration and recrystallized from an appropriate solvent.

*General Procedure E*. *N*-(Benzyl carbamothioyl)-substituted-2-hydroxy-benzamides **13a**, **b**, **e**, **f**, and **g** (0.5 mmol) were suspended in acetic acid (3 mL) in a 25 mL round-bottomed flask. The reaction mixture was heated to reflux for 2 hours then; the acetic acid was evaporated off under reduced pressure. The oily reaction mixture was triturated with minimal diethyl ether and the resulting solid products **15a**, **b**, **e**, **f**, and **g** were collected by vacuum filtration and recrystallized from an appropriate solvent.

Products **15a**, **b**, **e**, **f**, and **g** prepared in this procedure gave identical mp, IR, ^1^H NMR and ^13^C NMR to the analogues prepared from compound **14** with comparable yields (Scheme 3).

*2-(Benzyl amino)-4H-benz[e]-1,3-oxazin-4-one* ***15a***. 2-(Methylthio)-4*H*-benz[*e*]-1,3-oxazin-4-one **14a** was allowed to react with benzyl amine according to general procedure D. The crude solid was collected and recrystallized from ethanol to give **15a** (75% yield), mp 210°C. *ν*_max_ (KBr)/cm^−1^ 3065, 2872 (N–H), 1681 (C=O), 1635 (C=C), 1460 (C=N); ^1^H NMR (d_6_-DMSO) *δ* 8.70 (bs, 1H, N–H), 7.90 (d, 1H, *J* = 7.5 Hz, H-5), 7.70 (t, 1H, *J* = 7.5 Hz, H-6), 7.60–7.30 (m, 7H, ArH, H-7, H-8), 4.50 (s, 2H, H-9); ^13^C NMR (d_6_-DMSO) *δ* 165.4 (C-4), 154.9 (C-2), 151.6 (C-8a), 137.6 (C-1′), 135.2 (C-7), 127.7 (C-2′), 126.8 (C-4′), 126.4 (C-3′), 124.3 (C-5), 124.7 (C-6), 124.5 (C-8), 117.0 (C-4a), 43.8 (C-9); Anal. Calcd. For C_15_H_12_N_2_O_2_: C, 71.42; H, 4.79; N, 11.10. Found: C, 71.65; H, 4.95; N, 11.25.

*2-(Benzyl amino)-8-methyl-4H-benz[e]-1,3-oxazin-4-one* ***15b***. 8-Methyl-2-(methylthio)-4*H*-benz[*e*]-1,3-oxazin-4-one **14b** was allowed to react with benzyl amine according to general procedure D. The crude solid was collected and recrystallized from ethyl acetate to give **15b** (72% yield), mp 257–258°C. *ν*_max_ (KBr)/cm^−1^ 3062, 2883 (N–H), 1678 (C=O), 1639 (C=C), 1482 (C=N); ^1^H NMR (d_6_-DMSO) *δ* 8.80 (bs, 1H, N–H), 7.70 (d, 1H, *J* = 7.5 Hz, H-5), 7.50 (d, 1H, *J* = 7.5 Hz, H-7), 7.40–7.20 (m, ArH/H-6), 4.50 (s, 2H, H-9) 2.30 (s, 3H, 8-CH_3_); ^13^C NMR (d_6_-DMSO) *δ* 165.4 (C-4), 157.9 (C-2), 151.6 (C-8a), 137.6 (C-1′), 134.2 (C-7), 127.7 (C-2′), 126.8 (C-4′), 126.4 (C-3′), 124.3 (C-5), 124.7 (C-6), 124.5 (C-8), 117.0 (C-4a), 43.8 (C-9), 13.5 (CH_3_); Anal. Calcd. For C_16_H_14_N_2_O_2_: C, 72.16; H, 5.30; N, 10.52. Found: C, 72.10; H, 5.38; N, 10.25.

*2-(Benzyl amino)-8-phenyl-4H-benz[e]-1,3-oxazin-4-one* ***15c***. 8-Phenyl-2-(methylthio)-4*H*-benz[*e*]-1,3-oxazin-4-one **14c** was allowed to react with benzyl amine according to general procedure D. The crude solid was collected and recrystallized from ethanol to give **15c** (65% yield), mp 215°C. *ν*_max_ (KBr)/cm^−1^ 3040, 2860 (N–H), 1679 (C=O), 1635 (C=C), 1489 (C=N); ^1^H NMR (d_6_-DMSO) *δ* 9.20 (bs, 1H, N–H), 7.90 (d, 1H, *J* = 7.5 Hz, H-5), 7.70–7.20 (m, 11H, ArH/ArH/H-7), 7.90 (d, 1H, *J* = 3.0 Hz, H-6), 4.50 (s, 2H, H-9); ^13^C NMR (d_6_-DMSO) *δ* 165.1 (C-4), 157.9 (C-2), 151.2 (C-8a), 137.6 (C-1′), 134.7 (C-7), 134.1 (C-5), 129.2–125.8 (C-2′, C-4′ C-3′, C-8 C-9, C-10 C-11, C-12), 124.8 (C-6), 117.7 (C-4a), 43.9 (C-9); Anal. Calcd. For C_21_H_16_N_2_O_2_ 0.5H_2_O: C, 76.81; H, 4.91; N, 8.53. Found: C, 74.31; H, 4.99; N, 8.46.

*2-(Benzyl amino)-7-methoxy-4H-1,3-benzoxazin-4-one* ***15d***. 7-Methoxy-2-(methylthio)-4*H*-benz[*e*]-1,3-oxazin-4-one **14d** was allowed to react with benzylamine according to general procedure D. The crude solid was collected and recrystallised from toluene to give **15d** (83% yield), mp 234–236°C. *ν*_max_ (KBr)/cm^−1^ 3069–2891 (N–H), 1678 (C=O), 1619 (C=C), 1499 (C=N); ^1^H NMR (d_6_-DMSO) *δ* 9.00 (bs, 1H, N–H), 7.80 (d, 1H, *J* = 8.6 Hz, H-5), 7.30 (m, 5H, ArH), 6.90 (dd, 1H, *J*_H6,H8_ = 2.4 Hz, *J*_H6,H5_ = 8.6 Hz, H-6), 6.70 (d, 1H, *J* = 2.2 Hz, H-8), 4.50 (s, 2H, H-9), 3.90 (s, 3H, 7-OCH_3_); ^13^C NMR (d_6_-DMSO) *δ* 164.7 (C-4), 163.3 (C-7), 157.9 (C-2), 154.5 (C-8a), 137.6 (C-1′), 127.7 (C-5), 127.6 (C-2′), 126.7 (C-4′), 126.4 (C-3′), 112.2 (C-6), 110.6 (C-4a), 99.3 (C-8), 55.4 (OCH_3_), 43.7 (C-9); Anal. Calcd. For C_16_H_14_N_2_O_3_: C, 68.07; H, 5.00; N, 9.92. Found: C, 67.86; H, 4.89; N, 10.01.

*2-(Benzyl amino)-7-ethoxy-4H-1,3-benzoxazin-4-one* ***15e***. 7-Ethoxy-2-(methylthio)-4*H*-benz[*e*]-1,3-oxazin-4-one **14e** was allowed to react with benzylamine according to general procedure D. The crude solid was collected and recrystallized from toluene to give **15e** (80% yield), mp 214–216°C. *ν*_max_ (KBr)/cm^−1^ 3069–2827 (N–H), 1673 (C=O), 1600 (C=C), 1466 (C=N); ^1^H NMR (d_6_-DMSO) *δ* 9.00 (bs, 1H, N–H), 7.80 (d, 1H, *J* = 8.6 Hz, H-5), 7.30 (m, 5H, ArH), 6.90 (dd, 1H, *J*_H6,H8_ = 2.4 Hz, *J*_H6,H5_ = 8.6 Hz, H-6), 6.70 (d, 1H, *J* = 2.2 Hz, H-8), 4.50 (s, 2H, H-9), 4.20 (q, 2H, *J *= 5.6, CH_2_–O), 3.90 (s, 3H, 7-OCH_3_), 1.30 (t, 3H, *J *= 5.6, CH_3_); ^13^C NMR (d_6_-DMSO) *δ* 164.4 (C-4), 163.1 (C-7), 157.9 (C-2), 154.9 (C-8a), 137.8 (C-1′), 127.6 (C-5), 127.5 (C-2′), 126.7 (C-4′), 126.4 (C-3′), 112.6 (C-6), 110.3 (C-4a), 99.8 (C-8), 63.6 (CH_2_–O), 43.7 (C-9), 13.5 (CH_3_); Anal. Calcd. For C_17_H_16_N_2_O_3_: C, 68.91; H, 5.44; N, 9.45. Found: C, 69.11; H, 5.76; N, 9.28.

*2-(Benzyl amino)-7-hydroxy-4H-1,3-benzoxazin-4-one* ***15f***. 7-Hydroxy-2-(methylthio)-4*H*-benz[*e*]-1,3-oxazin-4-one **14f** was allowed to react with benzyl amine according to general procedure D. The crude solid was collected and recrystallized from ethanol to give **15f** (77% yield), mp decomp 262°C. *ν*_max_ (KBr)/cm^−1^ 3300–3058 (O–H), 3058–2851 (N–H), 1667s (C=O), 1607 m (C=C), 1543s (C=N); ^1^H NMR (d_6_-DMSO) *δ* 8.70 (bs, 1H, N–H), 7.50 (d, 1H, *J* = 8.6 Hz, H-5), 7.30–7.20 (m, 6H, ArH/7-OH), 6.50 (d, 1H, *J* = 8.6 Hz, H-6), 6.30 (s, 1H, H-8), 4.50 (s, 2H, H-9); ^13^C NMR (d_6_-DMSO) *δ* 169.6 (C-4), 166.1 (C-7), 158.1 (C-2), 155.8 (C-8a), 138.5 (C-1′), 128.3 (C-2′), 127.7 (C-4′), 127.2 (C-3′), 127.0 (C-5), 116.1 (C-6), 104.9 (C-4a), 100.6 (C-8), 43.7 (C-9); Anal. Calcd. For C_15_H_12_N_2_O_3_: C, 67.16; H, 4.51; N, 10.44. Found: C, 67.14; H, 4.34; N, 10.49.

*2-(Benzyl amino)-7-hydroxy-8-methyl-4H-1,3-benzoxazin-4-one* ***15g***. 7-Hydroxy-8-methyl-2-(methylthio)-4*H*-benz[*e*]-1,3-oxazin-4-one **14g** was allowed to react with benzylamine according to general procedure D. The crude solid was collected and recrystallised from ethanol to give **15g** (74% yield), mp 245–247°C. *ν*_max_ (KBr)/cm^−1^ 3300–2860 (O–H), 3058–2851 (N–H), 1678 (C=O), 1608 m (C=C), 1549s (C=N); ^1^H NMR (d_6_-DMSO) *δ* 8.70 (bs, 1H, N–H), 7.60 (d, 1H, *J* = 8.6 Hz, H-5), 7.30–7.20 (m, 6H, H-2′/H-3′/H-4′/7-OH), 6.80 (d, 1H, *J* = 8.6 Hz, H-6), 4.50 (s, 2H, H-9); ^13^C NMR (d_6_-DMSO) *δ* 165.2 (C-4), 159.6 (C-7), 157.8 (C-2), 152.5 (C-8a), 137.8 (C-1′), 127.6 (C-2′), 126.7 (C-4′), 126.4 (C-3′), 124.2 (C-5), 112.0 (C-6), 109.4 (C-8), 108.1 (C-4a), 43.7 (C-9), 6.8 (8-CH_3_); Anal. Calcd. For C_16_H_14_N_2_O_3_: C, 68.07; H, 5.00; N, 9.92. Found: C, 67.86; H, 5.28; N, 9.68.

*N-(2-(Benzylamino)-4-oxo-4H-benz[e][1,3]oxazin-7-yl)acetamide* ***15h***. In modification to the general procedure D, *N*-(2-(methylthio)-4-oxo-4*H*-benz[*e*][1,3]oxazin-7-yl)acetamide **14i** (0.26 g 1 mmol) was allowed to react with benzylamine (0.2 mL, 1 mmol) for 4 hours. The resulting solid was collected and recrystallised from acetonitrile to give **15 h** (0.1, 47%) as white crystals, mp 250–253°C. decomp. *ν*_max_ (KBr)/cm^−1^ 3284, 3234, 3215 (NH), 1669 (C=O); ^1^HNMR (200 MHz, 390 K, d_6_-DMSO) *δ* 10.28 (s, 1H, H-15), 8.86 (t, 1H, *J*_H9,H10_ = 4.7 Hz, H-9), 7.81–7.78 (m, 2H, H-8 and H-5), 7.37–7.26 (m, 6H, Ar, H-12–14 and H-6), 4.52 (d, 2H, *J*_H10,H9_ = 4.7 Hz, H-10), 2.10 (s, 3H, 17-CH_3_); ^13^C NMR (50 MHz, 390 K, d_6_-DMSO) *δ* 169.3 (C-16), 165.4 (C-4), 158.4 (C-8a), 154.2 (C-2), 144.3 (C-7), 138.2 (C-11), 128.4 (C-5), 127.5/127.3/127.2 (C-12/C-13/C-14), 115.7 (C-6), 112.1 (C-4a), 104.2 (C-8), 44.0 (C-10), 24.3 (C-17); Anal. Calcd. For C_17_H_15_N_3_O_3_: C, 66.01; H, 4.89; N, 13.58. Found: C, 65.93; H, 5.02; N, 13.52.

#### 4.1.14. 10-methyl-2,8-bis(methylthio)benzo[1,2-e:5,4-e′]bis([1,3]oxazine)-4,6-dione **24**

Following the previously reported [35], 10-methyl-2, 8-dithioxo-2, 3, 7, 8-tetrahydrobenzo[1,2-*e:*5,4-*e*′]bis([1,3]oxazine)-4,6-dione **18c** (0.74 g, 2.5 mmol) was allowed to react with methyl iodide (5.4 mL, 86.8 mmol) and NaHCO_3_ (3.0 g, 32 mmol) for 2 hrs. The resulting yellow solid **24** (0.78 g, 98%) is collected and used without further purification. mp > 300°C decomp. *ν*_max_ (KBr)/cm^−1^ 1623 (C=O), 1548 (C=N); ^1^H NMR (200 MHz, 390 K, d_6_-DMSO) *δ* 8.27 (s, 1H, H-5), 2.64 (s, 6H, H-3′and H-9′), 2.33 (s, 3H, H-10′). ^13^C NMR (50 MHz, 390 K, d_6_-DMSO) *δ* 173.0 (C-2,8), 160.6 (C-4,6), 155.3 (C-6a, 9a), 124.0 (C-5) 123.6 (C-10), 115.2 (C-4a, 5a), 13.4 (C-3′, 9′), 6.7 (C-10′).

#### 4.1.15. 2,8-Bis(benzylamino)-10-methylbenzo[1,2-e:5,4-e′]bis([1,3]oxazine)-4,6-dione **26**

In slight modification to the general procedure D,10-methyl-2,8-bis(methylthio) benzo[1,2-*e:*5,4-*e*′]bis([1,3]oxazine)-4,6-dione **24** (0.29 g, 1 mmol) was allowed to react with benzyl amine 0.4 mL, (2 mmol) for 16 hours. The resulting solid was collected and recrystallised from DMSO/water to give **26** (0.25 g, 56%) as an off white solid, mp 285–288°C decomp. *ν*_max_ (KBr)/cm^−1^ 3394, 3202, 3029 (NH), 1681, 1635 (C=O); ^1^HNMR (200 MHz, 340 K, d_6_-DMSO) *δ* 8.90 (bs, 2H, H-11,17), 8.27 (s, 1H, H-5), 7.43–7.24 (m, 10H 2 x Ar, H-14–16 and H-20–22), 4.58 (s, 4H, H-12,18), 2.31 (s, 3H, H-10′); ^13^C NMR (50 MHz, 340 K, d_6_-DMSO) *δ* 164.0 (C-4), 164.0 (C-9a,10a), 157.6 (C-2), 137.2 (C-13,19), 127.7, 126.9, 126.6, 122.5 (C-15,21, C-14,20 and C-16,22), 113.9 (C-10), 115.5 (C-4a,5a), 44.0 (C-12,17), 6.6 (C-10′); Anal. Calcd. For C_25_H_20_N_4_O_4_ *·*2H_2_O: C, 63.02; H, 5.08; N, 11.76. Found: C, 63.22; H, 4.99; N, 11.96.

#### 4.1.16. Synthesis of Substituted-1,3-benzoxazine-diones **22a–h** and **25**

*General Procedure F.* The appropriate substituted methylthio-1,3-benzoxazine 2.5 mmol was hydrolysed with 10 mL hydrochloric acid (10%) at 80°C for 4 hours. At the completion of the reaction, the reaction mixture was washed with R.O water, filtered, and recrystallised from an appropriate solvent. 

Products **22a–h** were used in the synthesis of products **23a–h** with no further purification.

*2H-Benz[e]-1,3-oxazin-2,4(3H)-dione* ***22a***. 2-(Methylthio)-4*H*-benz[*e*]-1,3-oxazin-4-one **14a** was allowed to react with hydrochloric acid (10%) according to general procedure F. The crude solid was collected and recrystallised from ethanol to give **22a** (75% yield), mp 228°C. (lit. [19, 20] 229-230°C). *ν*_max_ (KBr)/cm^−1^ 3179, 2877 (N–H), 1771 (C=O), 1690 (C=O), 1610 (C=C); ^1^H NMR (200 MHz, d_6_-DMSO) *δ* 7.90 (d, 1H, *J* = 7.5 Hz, H-5), 7.80 (t, 1H, *J* = 7.5 Hz, H-6), 7.40 (m, 2H, H-7/H-8); ^13^C NMR (50 MHz, d_6_-DMSO) *δ* 161.6 (C-4), 153.7 (C-2), 147.6 (C-8a), 136.2 (C-7), 126.9 (C-5), 125.2 (C-6), 116.5 (C-8), 114.6 (C-4a).

*8-Methyl-2H-benz[e]-1,3-oxazin-2,4(3H)-dione* ***22b***. 8-Methyl-2-(methylthio)-4*H*-benz[*e*]-1,3-oxazin-4-one **14b** was allowed to react with hydrochloric acid (10%) according to general procedure F. The crude solid was collected and recrystallised from ethanol to give **22b** (85% yield), mp 210°C (lit. [19, 20] 210–212°C). *ν*_max_ (KBr)/cm^−1^ 3221, 2845 (N–H), 1746 (C=O), 1717 (C=O), 1614 (C=C); ^1^H NMR (200 MHz, d_6_-DMSO) *δ* 8.80 (bs, 1H, N–H), 7.70 (d, 1H, *J* = 7.5 Hz, H-5), 7.60 (d, 1H, *J* = 7.5 Hz, H-7), 7.30–7.20 (t, 1H, *J* = 7.1 Hz, H-6), 2.30 (s, 3H, 8-CH_3_); ^13^C NMR (50 MHz, d_6_-DMSO) *δ* 161.6 (C-4), 151.9 (C-2), 147.3 (C-8a), 136.9 (C-7), 125.5 (C-8), 124.6 (C-5), 124.4 (C-6), 114.4 (C-4a), 13.8 (CH_3_).

*7-Methoxy-2H-benz[e]-1,3-oxazin-2,4(3H)-dione* ***22c***. 7-Methoxy-2-(methylthio)-4*H*-benz[*e*]-1,3-oxazin-4-one **14d** was allowed to react with hydrochloric acid (10%) according to general procedure F. The crude solid was collected and recrystallised from ethyl acetate to give **22c** (78% yield), mp 213°C. *ν*_max_ (KBr)/cm^−1^ 3203, 2930 (N–H), 1771 (C=O), 1723 (C=O), 1620 (C=C); ^1^H NMR (200 MHz, d_6_-DMSO) *δ* 12.10 (s, 1H, N–H), 7.90 (d, 1H, *J* = 8.3 Hz, 5-H), 7.10 (s, 1H, 8-H), 7.00 (d, 1H, *J* = 8.3 Hz, 6-H), 3.0 (s, 3H, 7-OCH_3_); ^13^C NMR (50 MHz, d_6_-DMSO) *δ* 160.6 (C-4), 157.9 (C-2), 155.4 (C-7), 151.6 (C-8a), 128.2 (C-5), 113.2 (C-6), 107.1 (C-4a), 100.8 (C-8), 56.5 (7-OCH_3_); Anal. Calcd. For C_9_H_7_NO_4_: C, 55.96; H, 3.65; N, 7.25. Found: C, 55.75; H, 3.45; N, 7.55.

*7-Ethoxy-2H-benz[e]-1,3-oxazin-2,4(3H)-dione* ***22d***. 7-Ethoxy-2-(methylthio)-4*H*-benz[*e*]-1,3-oxazin-4-one **14e** was allowed to react with hydrochloric acid (10%) according to general procedure F. The crude solid was collected and recrystallised from ethyl acetate to give **22e** (75% yield), mp 225–227°C. *ν*_max_ (KBr)/cm^−1^ 3157–2862 (N–H), 1771 (C=O), 1698 (C=O), 1620 (C=C); ^1^H NMR (200 MHz, d_6_-DMSO) *δ* 11.90 (bs, 1H, N–H), 7.80 (d, 1H, *J* = 8.6 Hz, H-5), 6.90 (m, 2H, H-6/H-8), 4.10 (q, 2H, *J *= 6.8 Hz, O–CH_2_), 1.30 (t, 3H, *J *= 6.8 Hz, CH_3_); ^13^C NMR (50 MHz, d_6_-DMSO) *δ* 164.6 (C-4), 160.9 (C-2), 155.4 (C-7), 147.6 (C-8a), 128.2 (C-5), 113.2 (C-6), 107.1 (C-4a), 100.8 (C-8), 64.4 (CH_2_–O), 14.3 (CH_3_); Anal. Calcd. For C_10_H_9_NO_4_: C, 57.97; H, 4.38; N, 6.76. Found: C, 57.86; H, 4.45; N, 6.55.

*7-Hydroxy-2H-benz[e]-1,3-oxazin-2,4(3H)-dione* ***22e***. 7-Hydroxy-2-(methylthio)-4*H*-benz[*e*]-1,3-oxazin-4-one **14f** was allowed to react with hydrochloric acid (10%) according to general procedure F. The crude solid was collected and recrystallised from ethanol to give **22e** (65% yield), mp 245°C (Lit [39] 310). *ν*_max_ (KBr)/cm^−1^ 3200–2700 (O–H), 3078, 2929 (N–H), 1780 (C=O), 1688 (C=O), 1616 (C=C); ^1^H NMR (200 MHz, d_6_-DMSO) *δ* 11.80 (s, 1H, N–H), 11.00 (s, 1H, 7-O–H), 7.70 (d, 1H, *J* = 8.6 Hz, H-5), 6.80 (d, 1H, *J* = 8.6 Hz, H-6), 6.60 (s, 1H, H-8); ^13^C NMR (50 MHz, d_6_-DMSO) *δ* 164.6 (C-4), 160.9 (C-2), 155.4 (C-7), 147.6 (C-8a), 128.6 (C-5), 113.8 (C-6), 105.9 (C-4a), 101.8 (C-8); Anal. Calcd. For C_8_H_5_NO_4_: C, 53.64; H, 2.81; N, 7.82. Found: C, 53.75; H, 2.45; N, 7.55.

*7-Hydroxy-8-methyl-2H-benz[e]-1,3-oxazin-2,4(3H)-dione* ***22f***. 7-Hydroxy-8-methyl-2-(methylthio)-4*H*-benz[*e*]-1,3-oxazin-4-one **14g** was allowed to react with hydrochloric acid (10%) according to general procedure F. The crude solid was collected and recrystallised from ethanol to give **22f** (70% yield), mp 250°C decomp. *ν*_max_ (KBr)/cm^−1^ 3250, 2900 (O–H), 3188, 2956 (N–H), 1771 (C=O), 1713 (C=O), 1619 (C=C); ^1^H NMR (200 MHz, d_6_-DMSO) *δ* 11.70 (s, 1H, N–H), 10.10 (s, 1H, 7-O–H), 7.60 (d, 1H, *J* = 8.4 Hz, H-5), 6.80 (d, 1H, *J* = 8.4 Hz, H-6), 2.10 (s, 3H, 8-CH_3_); ^13^C NMR (50 MHz, d_6_-DMSO) *δ* 162.4 (C-4), 161.5 (C-2), 153.4 (C-7), 147.9 (C-8a), 125.4 (C-5), 112.6 (C-6), 110.8 (C-4a), 106.0 (C-8), 8.1 (8-CH_3_); Anal. Calcd. For C_9_H_7_NO_4_: C, 55.96; H, 3.65; N, 7.25. Found: C, 55.75; H, 3.45; N, 7.55.

*N-(2,4-Dioxo-3,4-dihydro-2H-benz[e][1,3]oxazin-7-yl)acetamide* ***22g***. In slight modification to the general procedure F, *N*-(2-(methylthio)-4-oxo-4*H*-benz[*e*][1,3]oxazin-7-yl) acetamide **14i** (0.63 g, 2.5 mmol) was allowed to reflux with water (10 mL) for 2 hours. The resulting crude solid was filtered, and recrystallised from methanol to give **22 g** (0.52 g, 95%) as light grey solid, mp 284–287°C (Lit [39]. 310–14° decomp.). *ν*_max_ (KBr)/cm^−1^ 3350, 3050 (NH), 1758, 1704 (C=O); ^1^HNMR (200 MHz, 340 K, d_6_-DMSO) *δ* 11.80 (bs, 1H, 3-NH), 10.57 (s, 1H, 9-NH), 7.83 (d, 1H, *J*_H5,H6_ = 8.6 Hz, H-5), 7.72 (d, 1H, *J*_H8,H6_ = 1.6 Hz, H-8), 7.45 (dd, 1H, *J*_H6,H5_ = 8.6 Hz, *J*_H6,H8_ = 1.6 Hz, H-6), 2.11 (s, 3H, 11-CH_3_); ^13^C NMR (50 MHz, 340 K, d_6_-DMSO) *δ* 169.0 (C-10), 160.3 (C-4), 154.2 (C-2), 147.1, 145.7 (C-8a, C-7), 127.3 (C-5), 115.3 (C-6), 108.6 (C-4a), 104.6 (C-8), 23.5 (C-11). Anal. Calcd. For C_10_H_8_N_2_O_4_: C, 54.55; H, 3.66; N, 12.72. Found: C, 54.49; H, 3.77; N, 12.69.

*7-Amino-2H-benz[e][1,3]oxazine-2,4(3H)-dione* ***22h***. In modification to the general procedure F,* N*-(2-(methylthio)-4-oxo-4*H*-benzo[*e*][1,3]oxazin-7-yl) acetamide **14h** (0.63 g, 2.5 mmol) was allowed to react in the presence of hydrochloric acid (15 mL, 40%) for 4 hours. At the completion of the reaction, the reaction mixture was neutralized by NaHCO_3_, filtered and recrystallised from ethanol to give **22h** (0.34 g, 79%), mp 284–287°C decomp. *ν*_max_ (KBr)/cm^−1^ 3479, 3372 (NH), 2843 (NH), 1752, 1708 (C=O); ^1^HNMR (200 MHz, 340 K, d_6_-DMSO) *δ* 11.49 (s, 1H, 3-NH), 7.54 (d, 1H, *J*_H5,H6_ = 8.4 Hz, H-5), 6.52 (m, 3H, H-6 and 7-NH_2_), 6.31 (s, 1H, H-8).^13^C NMR (50 MHz, 340 K, d_6_-DMSO) *δ* 160.8 (C-4), 156.0, 155.6 (C-2, C-7), 147.9 (C-8a), 127.8 (C-5), 111.4 (C-6), 101.5 (C-4a), 97.3 (C-8). Compound **22h** was used for the synthesis of compound **23g** with no further purification.

*10-Methyl-2H,6H-[1,3]oxazino[5,6-g][1,3]benzoxazine-2,4,6,8(3H,7H)-tetrone* ***25***. 10-Methyl-2,8-bis(methylthio)benzo[1,2-*e:*5,4-*e*′]bis([1,3]oxazine)-4,6-dione **24** (0.74 g, 2.5 mmol) was allowed to react with hydrochloric acid (10%) for 4 hours according to the general procedure F. The resulting solid was recrystallised from DMF to give **25** (0.55 g, 85%) as off white crystals. mp > 300°C decomp. *ν*_max_ (KBr)/cm^−1^ 3183, 3119, 3049 (NH), 1784, 1714 (C=O), 1616 (C=C); ^1^HNMR (200 MHz, 340 K, d_6_-DMSO) *δ* 12.18 (bs, 2H, 3,7-NH), 8.26 (s, 1H, H-5), 2.31 (s, 3H, H-10′); ^13^C NMR (50 MHz, 340 K, d_6_-DMSO) *δ* 159.7 (C-4,6), 155.4 (C-2,8), 145.9 (C-9a,10a), 123.3 (C-5), 113.0 (C-10), 111.3 (C-4a,5a), 7.3 (C-10′); Anal. Calcd. For C_11_H_6_N_2_O_6_: C, 50.39; H, 2.31; N, 10.68. Found: C, 50.54; H, 2.42; N, 10.74.

#### 4.1.17. Synthesis of N-(Benzyl carbamoyl)-2-hydroxy-substituted-benzamide **23a–g**

*General Procedure G.* The appropriate 2-dione-1,3-benzoxazines **22a–b** and **22d–i** (2.5 mmol) were suspended in dry 1,4-dioxane (10 mL) in a 50 mL round-bottomed flask. Benzyl amine (12.5 mmol) was then added dropwise, directly from the pipette, with stirring, and then the reaction mixture was heated to reflux for 4 hours. At the completion of the reaction, it evaporated to dryness under reduced pressure and triturated with minimal diethyl ether. The resulting solid was collected by vacuum filtration and recrystallised from an appropriate solvent. 

*N-(Benzyl carbamoyl)-2-hydroxybenzamide* ***23a***. 2H-Benz[*e*]-1,3-oxazin-2,4(3*H*)-dione **22a** was allowed to react with benzyl amine according to general procedure F to give **23a** which was recrystallised from ethanol (70% yield), mp211–213°C. *ν*_max_ (KBr)/cm^−1^ 3340–2940 (O–H), 3230–3161 (N–H), 1687 (C=O), 1648 (C=O); ^1^H NMR (200 MHz, d_6_-DMSO) *δ* 11.75 (bs, 1H, H-2′), 10.40 (bs, 1H, 1-OH), 9.05 (t, 1H, *J* = 5.6 Hz, 4′-NH), 7.90 (d, 1H, *J* = 7.7 Hz, H-3), 7.50 (m, 1H, H-5), 7.30–7.20 (m, 5H, ArH), 7.05–6.95 (m, 2H, H-4/H-6), 4.40 (d, 2H, *J* = 5.2 Hz, H-5′); ^13^C NMR (50 MHz, d_6_-DMSO) *δ* 166.3 (C-1′), 158.7 (C-2), 153.1 (C-3′), 137.2 (C-6′), 133.7 (C-4), 130.8 (C-6), 128.4 (C-8′), 127.3 (C-7′), 127.0 (C-9′), 119.9 (C-1), 117.2 (C-5/C-3), 42.8 (C-5′); Anal. Calcd. For C_15_H_14_N_2_O_3_: C, 66.66; H, 5.22; N, 10.36. Found: C, 66.74; H, 5.36; N, 10.29.

*N-(Benzyl carbamoyl)-2-hydroxy-3-methylbenzamide* ***23b***. 8-Methyl-2*H*-benz[*e*]-1,3-oxazin-2,4(3*H*)-dione **22b** was allowed to react with benzyl amine according to general procedure G to give **23b** which was recrystallised from ethanol (65% yield), mp 220–223°C. *ν*_max_ (KBr)/cm^−1^ 3337–2946 (O–H), 3249 (N–H), 1696 (C=O), 1642 (C=O); ^1^H NMR (200 MHz, d_6_-DMSO) *δ* 11.30 (bs, 1H, H-2′), 10.70 (bs, 1H, 1-OH), 8.90 (t, 1H, *J* = 5.6 Hz, 4′-NH), 7.80 (d, 1H, *J* = 7.5 Hz, H-6), 7.40–7.20 (m, 6H, ArH and H-4), 6.80 (t, 1H, *J* = 7.5 Hz, H-5), 7.00–6.90 (m, 2H, H-4 and H-6), 4.60 (d, 2H, *J* = 5.2 Hz, H-5′), 2.20 (s, 3H, CH_3_); ^13^C NMR (50 MHz, d_6_-DMSO) *δ* 167.9 (C-1′), 159.7 (C-2), 154.9 (C-3′), 137.2 (C-6′), 135.0 (C-4), 128.4 (C-8′), 127.3 (C-7′), 127.0 (C-9′), 126.3 (C-3), 125.5 (C-6), 119.0 (C-5), 115.1 (C-1), 42.9 (C-5′), 15.9 (CH_3_); Anal. Calcd. For C_16_H_16_N_2_O_3_: C, 67.59; H, 5.67; N, 9.85. Found: C, 67.64; H, 5.54; N, 9.87.

*N-(Benzyl carbamoyl)-4-ethoxy-2-hydroxybenzamide* ***23c***. 7-Ethoxy-2*H*-benz[*e*]-1,3-oxazin-2,4(3*H*)-dione **22d** was allowed to react with benzyl amine according to general procedure F to give **23c** which was recrystallised from ethanol (70% yield), mp 204–206°C. *ν*_max_ (KBr)/cm^−1^ 3333–2876 (O–H), 3225–3156 (N–H), 1692 (C=O), 1650 (C=O); ^1^H NMR (200 MHz, d_6_-DMSO) *δ* 11.90 (bs, 1H, H-2′), 10.20 (bs, 1H, 2-OH), 8.00 (t, 1H, *J* = 5.4 Hz, 4′-NH), 7.90 (d, 1H, *J* = 8.9 Hz, H-6), 7.30 (m, 5H, ArH), 6.60 (dd, 1H, *J*_ H-5,H-3_ = 2.6 Hz, *J*_ H-5,H-6_ = 8.9 Hz, H-5), 6.50 (d, 1H, *J* = 2.2 Hz, H-3), 4.40 (d, 2H, *J* = 6.0 Hz, H-5′), 4.10 (q, 2H, *J *=6.8 Hz, CH_2_–O), 1.30 (t, 3H, *J *= 6.8 Hz, CH_3_); ^13^C NMR (50 MHz, d_6_-DMSO) *δ* 166.1 (C-1′), 160.7 (C-4), 158.9 (C-2), 153.2 (C-3′), 137.2 (C-6′), 132.4 (C-6), 128.4 (C-8′), 127.3 (C-7′), 127.0 (C-9′), 110.5 (C-5), 109.4 (C-1), 101.7 (C-3), 64.6 (CH_2_–O), 42.8 (C-5′), 14.5 (CH_3_); Anal. Calcd. For C_17_H_18_N_2_O_4_: C, 64.96; H, 5.77; N, 8.91. Found: C, 65.03; H, 5.82; N, 8.84.

*N-(Benzyl carbamoyl)-2,4-dihydroxybenzamide* ***23d***. 7-Hydroxy-2*H*-benz[*e*]-1,3-oxazin-2,4(3*H*)-dione **22e** was allowed to react with benzyl amine according to general procedure G and gave **23d** which was recrystallised from ethanol (75% yield), mp 220°C. *ν*_max_ (KBr)/cm^−1^ 3200–2700 (O–H), 3118, 3033 (N–H), 1692 (C=O), 1665 (C=O); ^1^H NMR (200 MHz, d_6_-DMSO) *δ* 9.00 (t, 1H, *J* = 5.6 Hz, 4′-N–H), 7.70 (d, 1H, *J* = 7.6 Hz, H-6), 7.30 (bm, 6H, ArH, 2′-OH), 6.30 (s, 1H, H-3), 6.2 (d, 1H, *J* = 7.6 Hz, H-5), 5.60 (m, 2H, 4-OH and 2′-NH) 4.40 (d, 2H, *J* = 6.0 Hz, H-5′); ^13^C NMR (50 MHz, d_6_-DMSO) *δ* 163.5 (C-1′), 161.9 (C-4), 158.5 (C-2), 153.2 (C-3′), 138.5 (C-6′), 135.3 (C-6), 128.7 (C-8′), 127.7 (C-7′), 127.5 (C-9′), 109.2 (C-1), 102.9 (C-5), 107.9 (C-3), 48.2 (C-5′); Anal. Calcd. For C_15_H_14_N_2_O_4_: C, 59.59; H, 4,67; N, 9.27. Found: C, 59.88; H, 4.68; N, 9.59.

*N-(Benzyl carbamoyl)-2,4-dihydroxy-3-methylbenzamide* ***23e***. 7-Hydroxy-8-methyl-2*H*-benz[*e*]-1,3-oxazin-2,4(3*H*)-dione **22f** was allowed to react with benzylamine according to general procedure G to give **23g** which was recrystallised from ethyl acetate (65% yield), mp 230°C. *ν*_max_ (KBr)/cm^−1^ 3381, 3225 (O–H), 3020–2800 (N–H), 1687 (C=O), 1639 (C=O); ^1^H NMR (200 MHz, d_6_-DMSO) *δ* 11.00 (bs, 1H, 2′-NH), 9.80 (bs, 1H, 2-OH), 8.5 (t, 1H, *J* = 5.6 Hz, 4′-NH), 7.5 (d, 1H, *J* = 8.8 Hz; H-5), 7.4 (bm, 5H, ArH), 6.9 (d, 1H, *J* = 8.8 Hz; H-6), 4.50 (d, 2H, *J* = 6.0 Hz, H-5′), 2.00 (s, 3H, CH_3_); ^13^C NMR (50 MHz, d_6_-DMSO) *δ* 165.2 (C-1′), 162.9 (C-4), 159.3 (C-2), 157.2 (C-3′), 137.5 (C-6′), 128.7 (C-8′), 127.7 (C-7′), 127.5 (C-9′), 127.3 (C-6), 112.2 (C-1), 110.9 (C-3), 107.9 (C-5), 44.2 (C-5′), 8.6 (3-CH_3_); Anal. Calcd. For C_16_H_16_N_2_O_4_: C, 63.99; H, 5.37; N, 9.33. Found: C, 63.88; H, 5.68; N, 9.59.

*4-Acetamido-2-hydroxy-N-(phenylcarbamoyl)benzamide* ***23f***. N-(2,4-Dioxo-3,4-dihydro-2H-benz[*e*][1,3]oxazin-7-yl) acetamide **22 g** was allowed to react with benzyl amine according to general procedure G. The resulting solid was filtered and recrystallised from ethanol to give **23f** (0.25 g, 76%) mp 257–260°C. *ν*_max_ (KBr)/cm^−1^ 3500–3200 (OH), 3281, 3122 (NH), 1694, 1664 (C=O), 1613, 1559 (C=C); ^1^H NMR (200 MHz, 340 K, d_6_-DMSO) *δ* 11.63 (bs, 1H, 10-NH), 10.24, 10.06 (15-NH, 2-OH), (s, 1H, *J*_H10,H11_ = 5.4 Hz, 10-NH), 7.84 (d, 1H, *J*_H6,H5_ = 8.8 Hz, H-6), 7.58 (d, 1H, *J*_H3,H5_ = 1.8 Hz, H-3), 7.38–7.24 (m, 5H, ArH, H-13, H-14, H-15), 7.03 (dd, *J*_H5,H6_ = 8.8 Hz, *J*_H5,H3_ = 1.8 Hz, H-3), 4.45 (d, 2H, *J*_H11,H10_ = 5.9 Hz, H-11), 2.07 (s, 3H, H-17); ^13^C NMR (50 MHz, 300 K, d_6_-DMSO) *δ* 168.7 (C-16), 165.7 (C-7), 157.5 (C-2), 152.9 (C-9), 144.6 (C-4), 138.8 (C-12), 131.1 (C-6), 128.1, 126.9, 126.7 (C-14, C-13, C-15), 111.2 (C-1), 110.5 (C-5), 106.1 (C-3), 42.6 (C-11), 23.9 (C-17); Anal. Calcd. For C_17_H_17_N_3_O_4_: C, 62.38; H, 5.23; N, 12.84. Found: C, 62.44; H, 5.28; N, 12.90.

*4-Amino-N-(benzyl carbamoyl)-2-hydroxybenzamide* ***23g***. 7-Amino-2H-benz[*e*][1,3]oxazine-2,4(3*H*)-dione **22h** was allowed to react with benzylamine for 16 h in slight modification to general procedure G. The resulting solid was filtered and recrystallised from toluene to give** 23g** (0.15 g, 54%) as light brown solid, mp 208–211°C decomp. *ν*_max_ (KBr) cm^−1^ 3500–3200 (OH), 3489, 3390, 3330 (NH), 1686 1645 (C=O); ^1^H NMR (200 MHz, 340 K, d_6_-DMSO) *δ* 10.18 (bs, 1H, 2-OH), 9.00 (t, 1H, *J*_H10,H11_ = 5.1 Hz 10-NH), 7.65 (d, 1H, *J*_H6,H5_ = 8.6 Hz, H-6), 7.34–7.27 (m, 6H, Ar, H-13, H-14, H-15 and 8-NH exchangeable with D_2_O), 6.19–6.11 (m, 2H, H-5 and H-3), 5.88 (bs, 2H, 4-NH_2_), 4.42 (d, 2H, *J*_H11,H10_ = 5.1 Hz, H-11); ^13^C NMR (50 MHz, 300 K, d_6_-DMSO) *δ* 166.6 (C-7), 159.4 (C-2), 154.8, 153.3 (C-4,C-9), 139.0 (C-12), 131.7 (C-6), 128.0 (C-14), 126.9, 126.6 (C-15, C-12), 106.6 (C-5), 105.5 (C-1), 99.2 (C-3), 42.5 (C-11); Anal. Calcd. For C_15_H_15_N_3_O_3_: C, 63.15; H, 5.30; N, 14.73. Found: C, 62.78; H, 4.90; N, 14.30.

### 4.2. Antibacterial Assays

#### 4.2.1. Determination of Minimal Inhibitory Concentrations (MICs) of Novel Compounds

Minimal inhibitory concentrations (MICs) were determined on 7 different bacterial and 4 fungi cultures. The bacteria include *Escherichia coli* (FB5), *Acinetobacter baumannii* (ATCC19606), *Pseudomonas aeruginosa* (PAO9503), *Staphylococcus aureus* (FB13), *Bacillus subtilis* (PAO9503), *Streptococcus agalactiae* (FB31) *Mycobacterium smegmatis* (CON 21), and *Mycobacterium chlorophenolicum *(CON 24). The fungi include *Aspergillus niger*, *Rhizopus oryzae*, *Absidia corymbifera,* and *Alternaria alternata*. Each one of the different strains of bacteria were cultured into 10 mL of nutrient broth (NB) or malt extract broth for fungi and incubated overnight at 37°C except for *Mycobacterium smegmatis* at 30°C for 24 hours and *Mycobacterium chlorophenolicum* for 4 days. All fungal cultures were grown at 25°C for up to seven days except for *Alternaria alternate,* 3 days 35°C. The grown cultures were diluted (1/10 in NB or Malt extract broth) and incubated for a further 2 hours (to reach exponential phase) and then used in the MIC assay. Compound stock solutions of 10^4^ *μ*g/mL were made up in DMSO ensuring that a maximum of 30 *μ*L is used, otherwise inhibitory effects will be shown on some bacterial/fungal cultures). In sterile microcentrifuge tubes, varying amounts of exponential phase culture (0.1 mL) were added and NB (0.9 mL) to make up a total volume of 1 mL. Each culture was then incubated at 37°C overnight. Control tubes were made using DMSO without the addition of compound. At the completion of the incubation, the microcentrifuge tubes containing culture were vortexed and compared to their respective controls (without compound). Compounds which displayed an absence of turbidity lower than 50 *μ*g/mL were subject to further dilutions, while if there is growth (or turbidity) at a particular concentration then the value is recorded as the MIC. The dilution series was carried out in factors of 2 as recommended (i.e., at 200, 100, 50, 25, 12.5, 6.25, and 3.125 *μ*g mL^−1^ resp.). The MIC was determined by the absence of turbidity at the lowest concentration. 

#### 4.2.2. Agar Disk Diffusion Method

Compounds that were insoluble in DMSO or the NB were evaluated for their antimicrobial activity by agar diffusion assays. The surface of an NA or Malt agar plate was flood-inoculated with an overnight NB or malt broth culture of a particular culture adjusted to 10^8^ CFU/mL (10^8^ colony forming units per millimeter). Each disk contained a specific culture and sterile 12.7-mm paper disk (oxide) was placed onto the dry surface for each compound. The insoluble compound was resuspended in DMSO and a 20 *μ*L aliquot was impregnated onto the surface of a sterile paper disc including 20 *μ*L of DMSO control. The diameter of the zone of inhibition was measured after incubation for 18 hr and compared to the control zone (DMSO).

## Figures and Tables

**Table 1:** Broth dilution susceptibility MIC values for inhibition growth of bacteria (*μ*gmL^−1^).

Bacteria								
#	*P. aeruginosa *	*B. subtilis *	*S. aureus *	*A. baumannii *	*E. coli *	*S. agalactiae *	*M. smegmatis *	*M. chloro *
**9c**	200	200	>200	200	200	n/a	>200	50
**9d**	200	100	25	200	200	n/a	>200	25
**13c**	>300	>300	>300	>300	>300	>300	n/a	n/a
**13d**	200	>300	>300	200	>300	300	n/a	n/a
**13e**	>300	>300	300	>300	>300	>300	n/a	n/a
**13g**	>300	>300	>300	>300	>300	>300	n/a	n/a
**13j**	>300	>300	>300	>300	>300	200	n/a	n/a
**13l**	>300	>300	300	>300	>300	>300	n/a	n/a
**13m**	200	200	300	>300	>300	>300	n/a	n/a
**13n**	300	>300	300	300	>300	300	n/a	n/a
**20a**	>200	12.5	>200	>200	>200	n/a	>200	>200
**20c**	>200	25	>200	>200	>200	n/a	>200	>200
**21**	>200	25	200	>200	>200	n/a	100	50
**22g**	200	>200	>200	200	>200	n/a	>200	200

**Table 2:** Broth dilution susceptibility MFC values for inhibition growth of fungi (*μ*gmL^−1^).

Fungi				
#	*R. oryzae *	*A. niger *	*A. corymbifera *	*A. alternata *
**9c**	200	>200	>200	>200
**9d**	200	>200	100	>200
**22g**	>200	>200	>200	>200
**20a**	>200	>200	>200	>200
**20c**	>200	>200	>200	>200
**21**	>200	>200	200	200

**Table 3:** Results showing Disc diffusion susceptibility for the synthesised compounds.

Bacteria								
#	*P. aeruginosa *	*B. subtilis *	*S. aureus *	*A. baumannii *	*E. coli *	*S. agalactiae *	*M. smegmatis *	*M. chloro *
**8c**	S (2 mm)	R	R	R	R	n/a	S (2 mm)	R
**8d**	S (2 mm)	S (3 mm)	R	R	R	n/a	S (2 mm)	S (2 mm)
**9c**	S (2 mm)	S (2 mm)	R	R	R	n/a	S (2 mm)	S (2 mm)
**9d**	S (2 mm)	S (4 mm)	R	R	R	n/a	S (2 mm)	S (2 mm)
**13b**	R	S (6 mm)	S (5 mm)	R	R	S (5 mm)	n/a	n/a
**13k**	R	S (2 mm)	S (5 mm)	S (2 mm)	R	S (5 mm)	n/a	n/a
**13f**	R	S (2 mm)	S (5 mm)	S (2 mm)	R	S (5 mm)	n/a	n/a
**15d**	R	S (2 mm)	S (5 mm)	S (2 mm)	R	S (5 mm)	n/a	n/a
**15e**	R	R	R	R	R	R	n/a	n/a
**13o**	R	R	R	R	R	—	R	R
**13p**	R	R	R	R	R	n/a	S (2 mm)	R
**13q**	R	R	R	R	R	n/a	R	R
**20a**	R	S (2 mm)	R	R	R	n/a	R	R
**20c**	R	S (2 mm)	R	R	R	n/a	S (3 mm)	S (4 mm)
**21**	R	S (4 mm)	R	R	R	n/a	R	R
**22h**	R	R	R	R	R	n/a	S (3 mm)	S (3 mm)
**22i**	R	R	R	R	R	n/a	S (2 mm)	R
**23g**	R	R	R	R	R	n/a	R	R

**Table 4:** Disc diffusion susceptibility for the synthesised compounds.

Fungi				
#	*R. oryzae *	*A. niger *	*A. corymbifera *	*A. alternata *
**8c**	R	R	R	R
**8d**	S (2 mm)	S (3 mm)	S (5 mm)	R
**9c**	R	R	R	R
**9d**	R	R	R	R
**13p**	S (2 mm)	R	R	R
**13q**	S (2 mm)	S (2 mm)	S (3 mm)	R
**21c**	S (2 mm)	S (2 mm)	S (3 mm)	R
**20a**	R	R	R	R
**20c**	R	R	R	R
**22h**	R	R	R	R
**22i**	R	R	R	R
**25b**	R	R	R	R
